# Development and Validation of a Sensitive and Specific LC-MS/MS Method for IWR-1-Endo, a Wnt Signaling Inhibitor: Application to a Cerebral Microdialysis Study

**DOI:** 10.3390/molecules27175448

**Published:** 2022-08-25

**Authors:** Sreenath Nair, Abigail Davis, Olivia Campagne, John D. Schuetz, Clinton F. Stewart

**Affiliations:** Department of Pharmacy and Pharmaceutical Science, St. Jude Children’s Research Hospital, Memphis, TN 38105, USA

**Keywords:** IWR-1-endo, Wnt signaling inhibitor, LC-MS/MS, solid-phase extraction, pharmacokinetics, cerebral microdialysis, bioanalysis

## Abstract

IWR-1-endo, a small molecule that potently inhibits the Wnt/β-catenin signaling pathway by stabilizing the AXIN2 destruction complex, can inhibit drug efflux at the blood–brain barrier. To conduct murine cerebral microdialysis research, validated, sensitive, and reliable liquid chromatography–tandem mass spectrometry (LC-MS/MS) methods were used to determine IWR-1-endo concentration in the murine plasma and brain microdialysate. IWR-1-endo and the internal standard (ISTD) dabrafenib were extracted from murine plasma and microdialysate samples by a simple solid-phase extraction protocol performed on an Oasis HLB µElution plate. Chromatographic separation was executed on a Kinetex C_18_ (100A, 50 × 2.1 mm, 4 µm particle size) column with a binary gradient of water and acetonitrile, each having 0.1% formic acid, pumped at a flow rate of 0.6 mL/min. Detection by mass spectrometry was conducted in the positive selected reaction monitoring ion mode by monitoring mass transitions 410.40 > 344.10 (IWR-1-endo) and 520.40 > 307.20 (ISTD). The validated curve range of IWR-1-endo was 5–1000 ng/mL for the murine plasma method (r^2^ ≥ 0.99) and 0.5–500 ng/mL for the microdialysate method (r^2^ ≥ 0.99). The lower limit of quantification (LLOQ) was 5 ng/mL and 0.5 ng/mL for the murine plasma and microdialysate sample analysis method, respectively. Negligible matrix effects were observed in murine plasma and microdialysate samples. IWR-1-endo was extremely unstable in murine plasma. To improve the stability of IWR-1-endo, pH adjustments of 1.5 were introduced to murine plasma and microdialysate samples before sample storage and processing. With pH adjustment of 1.5 to the murine plasma and microdialysate samples, IWR-1-endo was stable across several tested conditions such as benchtop, autosampler, freeze–thaw, and long term at −80 °C. The LC-MS/MS methods were successfully applied to a murine pharmacokinetic and cerebral microdialysis study to characterize the unbound IWR-1-endo exposure in brain extracellular fluid and plasma.

## 1. Introduction

Group 3 medulloblastoma (MB) accounts for 25% of all MB and is considered the most aggressive form of this common malignant childhood brain tumor [1,2]. Patients with Group 3 MB have a poor prognosis, with a 5-year overall survival of less than 60% [1,2]. Thus, the development of novel therapeutic approaches is crucial for this MB subgroup.

Effective therapy for MB should overcome the blood–brain barrier (BBB) and blood–tumor barrier (BTB), as well as the efflux pumps associated with these barriers [3]. The ABC transporters are the main efflux transporters expressed at the BTB. Further, we previously showed that the ABCG2 transporter was highly expressed in Group 3 MB and inhibition of ABCG2 intensified antitumor activity [4]. Several studies have investigated the coadministration of efflux transporter inhibitors as a therapeutic strategy to improve penetration across both the BBB and BTB [3].

Recent studies show that inhibitors of the canonical Wnt/β-catenin signaling pathway can regulate the expression of ABC transporter expression in various cancers [5,6,7]. One of these therapeutic compounds, IWR-1-endo, is a small molecule that potently inhibits the Wnt/β-catenin signaling pathway by stabilizing the AXIN2 destruction complex [7,8]. IWR-1-endo also blocked doxorubicin efflux in a drug-resistant model of osteosarcoma [7]. An ABCG2 structural-based pharmacophore model provided the evidence to suggest that IWR-1-endo fits the ABCG2 pharmacophore and has the potential to block the efflux of anticancer drugs at the BBB [9]. Thus, to understand the in vivo central nervous system (CNS) penetration of IWR-1-endo, we performed cerebral microdialysis studies in murine models.

Performing these studies required that we have a sensitive and specific LC-MS/MS method for IWR-1-endo in murine plasma and microdialysates; however, to our knowledge, no bioanalytical methods for IWR-1-endo have been published. In this study, we reported LC-MS/MS methods for murine plasma and microdialysates that were developed for use in our murine cerebral microdialysis experiments.

## 2. Results and Discussion

### 2.1. Optimization of Mass Spectrometric and Chromatographic Conditions

**Figure 1:**
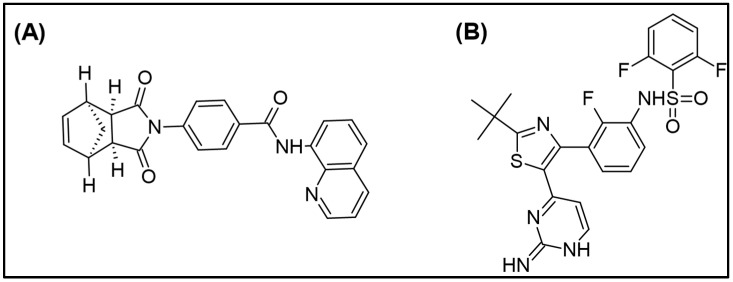
Structure of (**A**) IWR-1 endo and (**B**) ISTD, dabrafenib.

The compound IWR-1-endo (Figure 1A) is in the preclinical development stage, and no data are published on LC-MS/MS determination of IWR-1-endo in biological matrices. Thus, syringe pump infusion experiments were essential to identify the most sensitive precursor and fragment ion pair for IWR-1-endo and dabrafenib (ISTD; Figure 1B).

**Figure 2:**
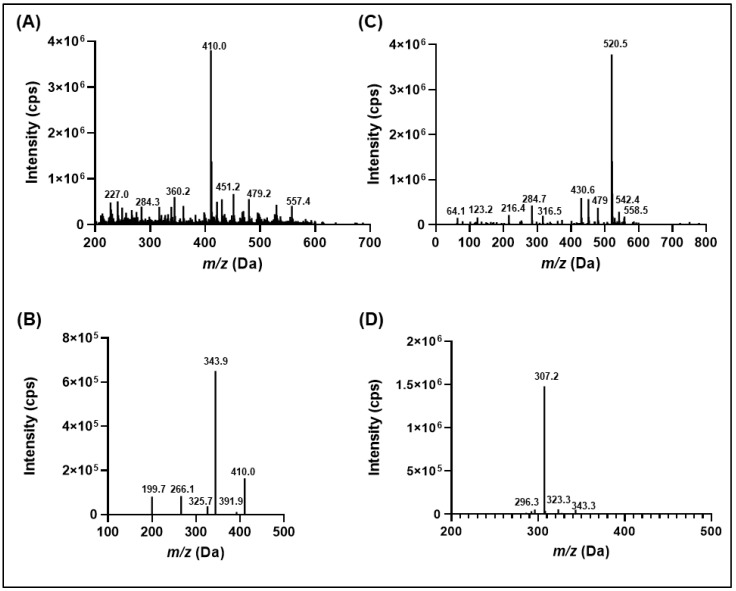
Representative full scan precursor (**A**) and product ion (**B**) scans for IWR-1-endo and corresponding ISTD, dabrafenib (**C**,**D**).

**Figure 3:**
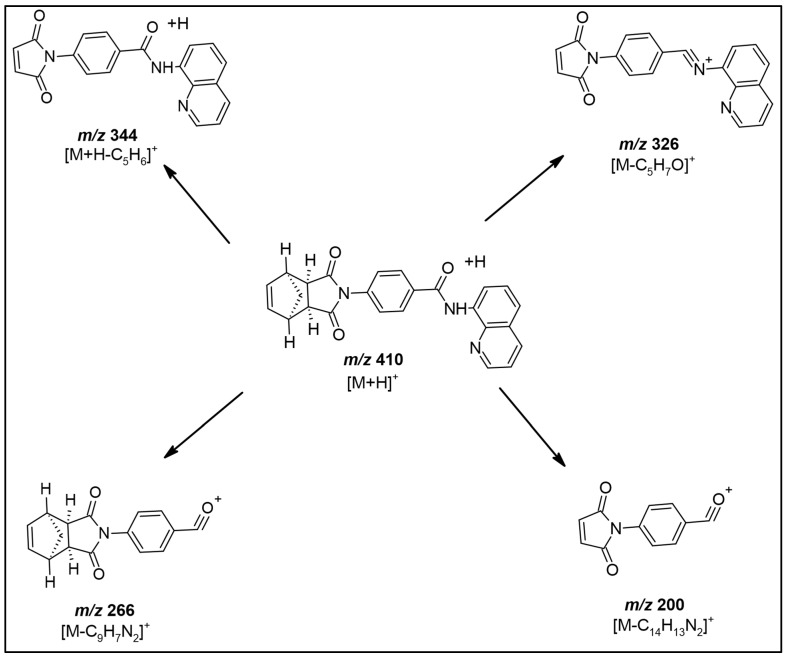
Proposed fragmentation pattern for the prominent product ions of IWR-1-endo.

Initially, IWR-1-endo prepared in methanol (0.001 mg/mL) was infused in both positive and negative ionization modes on an AB Sciex 4000 Q-TRAP mass spectrometer. IWR-1-endo could be easily protonated in the positive ion mode to generate the molecular ion peak, [M+H]^+^ at *m/z* 410.40. Next, the protonated precursor ion was fragmented using a collision energy of 25 eV to yield a high-intensity fragment ion at *m/z* 344.1. Other prominent fragment ion peaks were generated at *m/z* 383.4 and 274.2. Figure 2A–D demonstrates the typical precursor and product ion mass spectrum obtained for IWR-1-endo and ISTD under optimized mass spectrometric conditions in this study. Figure 3 depicts the proposed fragmentation pattern for IWR-1-endo at CE of 25 eV. The fragmentation pattern for dabrafenib has been discussed in a previous report [10].

The liquid chromatographic conditions were optimized to ensure better peak shape and separation of IWR-1-endo and ISTD, enhanced sensitivity for the analyte, and avoid matrix effect issues. Given the hydrophobic nature of IWR-1-endo (theoretical log *p*-value: 2.90), the initial method was developed using a reversed-phase C_18_ column on Shimadzu Nexera X2 HPLC equipment. By employing the gradient profiles described in Section 3.5 chromatographic conditions, acetonitrile/water and methanol/water mobile phase systems were compared, with the former showing a higher IWR-1-endo peak response and a lower background noise (Figure S1). Subsequently, the effect of acidic mobile phase additives, including formic acid (0.05 and 0.1%), acetic acid (0.05 and 0.1%), and liquid ammonia (0.05 and 0.1%), were investigated. Better peak shapes were obtained when adding 0.1% formic acid to both mobile phases A and B, which might be due to the ease of protonation in the acidic environment. Based on the above optimization, a gradient elution comprising an acetonitrile/water system modified with 0.1% formic acid pumped at a flow rate of 0.6 mL/min was employed for further experiments (Figure S1).

Next, the peak shapes on two analytical columns (i.e., Kinetex^®^ C_18_ analytical column (100°A, 50 × 2.1 mm, 2.6 µm particle size) and Kinetex^®^ C_8_ analytical column (100°A, 50 × 2.1 mm, 2.6 µm particle size)) from Phenomenex (Torrance, CA, USA) were compared. With the C_18_ column, the ISTD eluted closer to the retention time of the analyte, which helped address the matrix effect problems when working with microdialysate samples (Figure S2). Another important contributor to peak shape and analyte response was the injection volume, which was set at 2 µL and 10 µL for the murine plasma and microdialysate methods, respectively. A higher injection volume was used for the microdialysate method, given its lower curve range compared to that of the murine plasma method. Injection volumes above 10 µL not only affected the peak shape but also introduced matrix effects from the microdialysates. Lastly, a strong needle wash of methanol/acetonitrile/water/2-propanol (1:1:1:1; *v/v/v/v*) modified with 0.1% formic acid was used between runs to reduce carry-over of the analyte. The typical experimental retention times for IWR-1-endo and ISTD on the C_18_ column under the optimized chromatographic conditions were 1.72 ± 0.05 min and 1.65 ± 0.05 min, respectively. The total run time of this method was 5 min, which included a 1.75 min re-equilibration time for the column to return to initial chromatographic conditions between subsequent runs.

### 2.2. Optimization of Sample Preparation

The optimization of the sample pretreatment protocol was a vital part of this study since high method sensitivities were required for the LC-MS/MS methods developed in murine plasma and microdialysate samples. Initially, protein precipitation was tested with methanol and acetonitrile leading to low and inconsistent extraction recoveries for IWR-1-endo in murine plasma and microdialysate samples. As a result, solid-phase extraction (SPE) was considered, which was not only instrumental in improving overall extraction recovery from plasma and microdialysate samples but also providing consistent recoveries. Waters^®^ oasis method development 96-well µElution sorbent selection plate and HLB plate (2 mg sorbent per well, 30 µm) were evaluated for plasma and microdialysate sample cleanup. High recoveries and clean chromatograms for IWR-1-endo and ISTD were obtained by HLB µElution plates (Table S1). Additionally, the eluting capabilities of numerous solvent systems for extracting IWR-1-endo and ISTD from the SPE plates were investigated. Of these solvents, 140 µL of ACN modified with methanol (9:1, *v/v*) was able to disrupt all types of interactions in the case of IWR-1-endo and ISTD. Thus, it was used as an elution solvent for the final SPE extraction step. Lastly, dabrafenib was used as ISTD in the current assay as it did not interfere with the estimation of IWR-1-endo, and it resembled IWR-1-endo in terms of extractability from plasma and microdialysate samples.

### 2.3. Stability of IWR-1-Endo in Murine Plasma under Different StorageC

**Figure 4:**
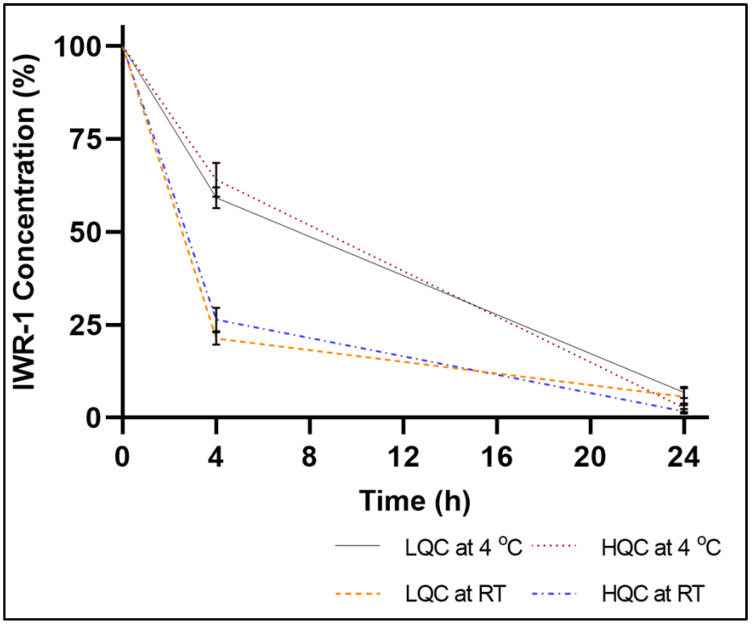
Time course of degradation of IWR-1 in murine plasma stored at 4 °C and room temperature over a period of 24 h (n = 3; mean ± S.D.). LQC: low-quality control; HQC: high-quality control; RT: room temperature.

During the prevalidation runs, short-term stability studies performed at RT and 4 °C, over a time course of 24 h showed significant IWR-1-endo degradation in K_2_EDTA-treated CD1 murine plasma (Figure 4). In a parallel experiment, IWR-1-endo quality control working solutions kept at RT and 4 °C had no stability-related issues, thus suggesting that IWR-1-endo was unstable in plasma. It has been previously demonstrated that the anticoagulant could impact the compound stability [11,12]. Therefore, we repeated the short-term stability studies with sodium-heparin-treated CD1 murine plasma. However, significant IWR-1-endo degradation was also observed in heparin-treated plasma for all the tested stability samples, similar to the K_2_EDTA plasma results (data not shown). Since matrix-related irreproducibility is more pronounced in heparin-treated plasma, we used K_2_EDTA as the anticoagulant for further optimization studies. We next tested the freeze–thaw stability as well as long-term stability of IWR-1-endo in CD1 murine plasma at −80 °C. As expected, a significant loss of drug stability was observed during both FTS and LTS studies.

To further identify whether the drug loss is due to non-specific binding (NSB) or biological sample instability, we subsequently performed STS and FTS studies in murine plasma with 0.5% Tween-80 solution to evaluate the drug’s adsorption to the surface of sample storage containers. The addition of Tween-80 to the biological matrix avoids NSB effects. However, the samples fortified with Tween-80 solutions show decreased IWR-1-endo concentrations for both STS and FTS studies, similar to the untreated QCs, suggesting instability of IWR-1-endo in murine plasma.

### 2.4. Addressing IWR-1-Endo Instability in Murine Plasma

Previous reports have suggested that the major biotransformation of drugs in plasma is enzymatic hydrolysis, which leads to drug degradation [13,14]. The major enzymes involved in this hydrolysis include carboxylesterases, acetylcholinesterase, cholinesterase, peptidases, and nucleases. We initially hypothesized that one of the above-mentioned matrix enzymes might be the cause of IWR-1-endo instability in murine plasma and microdialysates during sample storage and processing. Since we could not identify the actual enzyme responsible for the degradation of IWR-1-endo in the biological matrix, we tested several common enzyme inhibitors such as p-chloromercuribenzoate (A-esterase inhibitor), bis-(p-nitrophenyl) phosphate (B-esterase inhibitor), and sodium fluoride (acetylcholinesterase inhibitor). However, it was observed that IWR-1-endo was unstable in plasma despite the addition of enzyme inhibitors (Figure S3**)**. We finally resorted to pH adjustment since most enzymes are active over a narrow pH range [15]. We used several acidic and alkaline solutions to adjust murine plasma over a range of pH values (i.e., 1–11). The data suggested that IWR-1-endo was extremely stable at pH = 1.5, obtained by the addition of 200 µL dilute hydrochloric acid (0.1 N HCl) to the 20 µL of murine plasma and microdialysate samples after the microdialysis study sample collection (Figure S4).

### 2.5. Validation of the LC-MS/MS Methods

**Figure 5:**
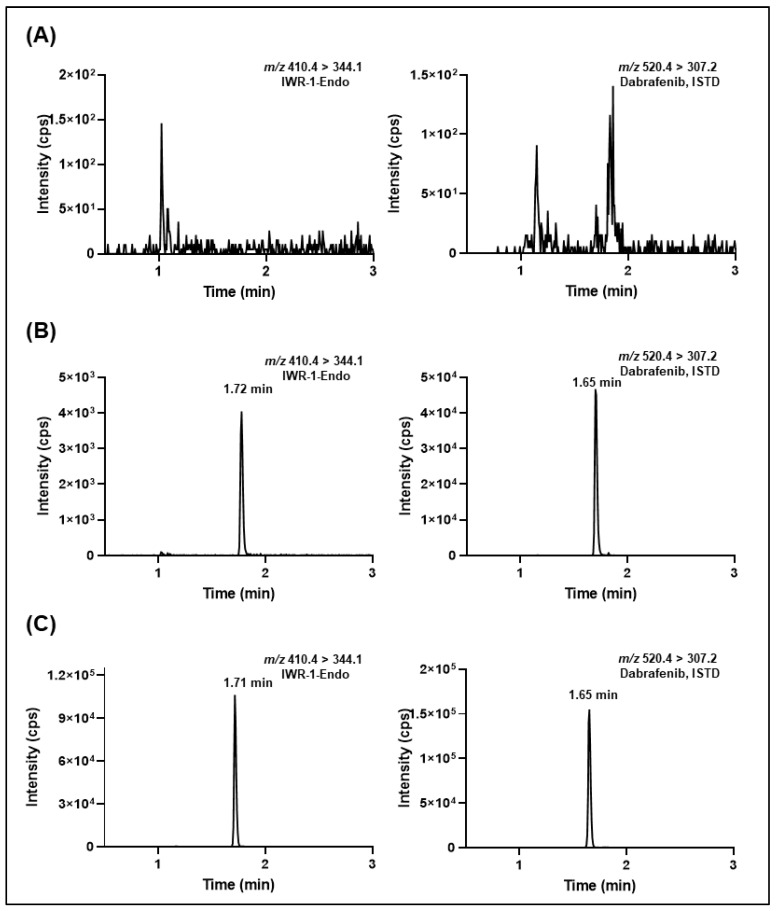
Representative extracted-ion chromatograms of (**A**) murine blank plasma, (**B**) IWR-1-endo-spiked murine plasma sample at LLOQ (5 ng/mL) with ISTD added, and (**C**) 1 h plasma sample from the microdialysis study (ISTD added) after administration of 30 mg/kg IWR-1-endo intraperitoneally.

**Figure 6:**
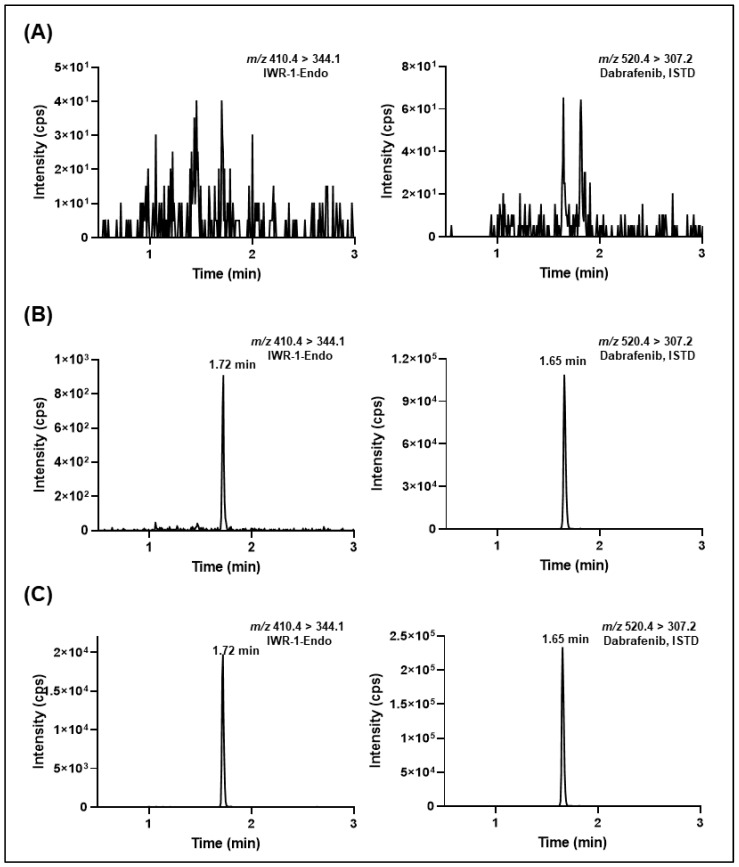
Representative extracted-ion chromatograms of (**A**) microdialysate blank, (**B**) IWR-1-endo-spiked microdialysate sample at LLOQ (0.5 ng/mL) with ISTD added, and (**C**) 1 h plasma sample from the microdialysis study (ISTD added) after administration of 30 mg/kg IWR-1-endo intraperitoneally.

Figure 5 and Figure 6 depict the typical SRM chromatograms for the double blank, lower limit of quantitation (LLOQ), and in vivo murine plasma and microdialysate study samples collected at 1 h after intraperitoneal administration of IWR-1-endo (30 mg/kg). No interfering peaks were found at the retention of IWR-1-endo or ISTD, suggesting acceptable selectivity of the developed methods. Additionally, a negligible carry-over effect was observed in double-blank samples after the injection of the upper limit of quantitation (ULOQ) samples for both the murine plasma and microdialysate methods This was mainly due to the use of a strong flushing solvent comprising methanol/acetonitrile/water/2-propanol (1:1:1:1; *v/v*), modified with 0.1% formic acid between the sample runs. The %C.V. observed during method validation for system suitability samples (injected at LLOQ concentrations) for mouse plasma and microdialysate sample analysis was ≤8% (n = 6). The typical retention time for IWR-1-endo was 1.72 ± 0.05 min, and the peak asymmetry obtained for IWR-1-endo was 1.02 ± 0.04 (n = 6).

Table 1 provides the concentration ranges, regression equations, and correlation coefficient results for the determination of IWR-1-endo in murine plasma and microdialysates. The calibration curves showed good linearity over the tested concentration ranges with r^2^ ≥ 0.9989 and 0.9990 for murine plasma and microdialysates, respectively (Figure S5). The LLOQ was 5 ng/mL and 0.5 ng/mL for IWR-1-endo in murine plasma and microdialysates, respectively, at which the S/N ratio was ≥10, and the accuracy (% relative error) was within ±20% with precision (R.S.D.) ≤20%. The LODs obtained were 0.50 ng/mL (C.V.: 29.48%, n = 6) and 0.20 ng/mL (C.V.: 20.01%, n = 6) for the murine plasma and microdialysate samples, correspondingly.

Table 2 and Table 3 illustrate the summary of the within-run and between-run precision and accuracy for IWR-1-endo in murine plasma and microdialysate samples, respectively, obtained during the three days of validation by analyzing the quality control samples. The relative error (RE) ranged from −0.86% to 9.26%, with RSD below 10.10% for the murine plasma method. The RE ranged from −8.86% to 0.21%, with RSD below 7.31% for the microdialysate method, indicating that the developed method was reliable and reproducible for IWR-1-endo quantification.

The matrix effect and recovery results in murine plasma and microdialysates are illustrated in Table 4. The mean MF for IWR-1-endo ranged from 0.99 to 1.02, with R.S.D. ≤ 2.55% for murine plasma and 1.05 to 1.13, with R.S.D. of ≤9.73 % for the microdialysate samples, respectively, indicating negligible matrix effect. The average extraction recoveries for IWR-1-endo in CD1 and CD1 nude murine plasma samples were 97.54% and 99.00%, respectively, with the R.S.D. ≤ 3.23% for all the QC samples analyzed. Likewise, the average extraction recoveries for IWR-1-endo in microdialysate samples was 88.76%, with the R.S.D. ≤ 10.00% for all the analyzed QC samples. The average extraction recoveries for ISTD in murine plasma and microdialysate samples were 103.51% and 94.94%, respectively, with the R.S.D. value ≤ 4.78% for all the analyzed QC samples.

The results of the stability assessments performed for IWR-1-endo-spiked in murine plasma and microdialysates under different experimental storage conditions are summarized in Table 5. IWR-1-endo was stable for 48 h in spiked murine plasma and microdialysate samples after short-term storage at 4 °C and room temperature, indicating that the samples were stable under the laboratory handling conditions. The stability of IWR-1-endo was confirmed in spiked murine plasma and microdialysate samples after long-term storage at −80 °C for 21 and 29 days, respectively, highlighting the reliability of the developed LC-MS/MS method to handle in vivo study samples. In addition, the extracted quality control samples for IWR-1-endo in murine plasma and microdialysates were stable in the autosampler for 48 h, indicating good post-extractive stability for the analyte. Further, no stability-related concerns for IWR-1-endo were observed for murine plasma and microdialysate samples undergoing three freeze–thaw cycles. When stored at −80 °C for 83 days, the primary stock solution of IWR-1-endo was stable with an R.S.D ≤ 2.30%.

### 2.6. Application to Cerebral Microdialysis Studies

**Figure 7:**
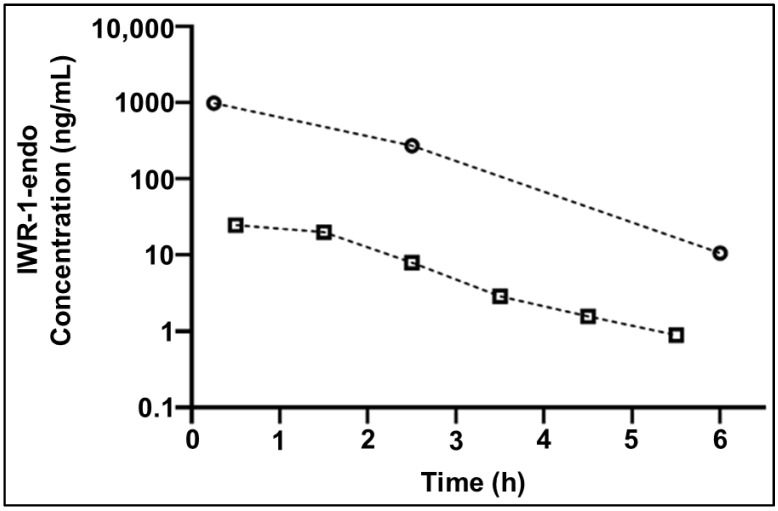
IWR-1-endo concentration–time profile in plasma and brain extracellular fluid (ECF) in a female non-tumor-bearing CD1 nude mouse. Open circles represent IWR-1-endo plasma concentrations, and open squares represent IWR-1-endo brain ECF concentrations.

IWR-1-endo concentrations were measured in murine plasma and Ringer’s solution collected in a female non-tumor-bearing mouse dosed with 30 mg/kg IWR-1-endo intraperitoneally (Figure 7). The microdialysis probe recovery was 61%. IWR-1-endo total plasma and unbound ECF exposures were calculated as the area under the concentration–time curve (AUC), using a noncompartmental pharmacokinetic analysis. IWR-1-endo total plasma and unbound ECF AUC were 1915 and 45.4 h·ng/mL, respectively, showing a total brain ECF to plasma partition coefficient of 0.024 for IWR-1-endo.

## 3. Materials and Methods

### 3.1. Reagents and Chemicals

The reference standard for IWR-1-endo (M.W.: 409.40 g/mol; M.F.: C_25_H_19_N_3_O_3;_ purity determined by LC-MS as 99.18%) was synthesized in-house by the Department of Chemical Biology and Therapeutics, St. Jude Children’s Research Hospital (Memphis, TN, USA) [8]. The ISTD dabrafenib (M.W.: 519.56 g/mol; M.F.: C_23_H_20_F_3_N_5_O_2_S_2_; purity determined by HPLC and TLC as 100%) was obtained from Cayman Chemical (Ann Arbor, MI, USA). Blank K_2_EDTA CD-1 murine plasma was purchased from BioIVT (Westbury, NY, USA). Laboratory-grade Ringer’s solution (98.97% H_2_0, 0.95% NaCl, 0.04 % KCl, 0.02 % CaCl_2_, and 0.02% NaHCO_3_) was obtained from Frey Scientific (Nashua, NH, USA) and used as the artificial microdialysate. Trappsol^®^ 1-hydroxypropyl-β-cyclodextrin (BCD), pharmaceutical grade, was purchased from Cyclodextrin Technologies Development (Gainesville, FL, USA). Optima^TM^ LC/MS-grade acetonitrile, Optima^TM^ LC/MS-grade methanol, ACS-grade dimethyl sulfoxide (DMSO), J. T. Baker^TM^ 1-methyl-2-pyrrolidinone (NMP), Medchemexpress Solutol HS-15, Fine Chemicals Biosciences d-α-tocopherol polyethylene glycol 1000 succinate (TPGS), MilliporeSigma^TM^ polyethylene glycol 400, and Honeywell Fluka™ formic acid for mass spectrometry were purchased from Fisher Scientific (Waltham, MA, USA). All other chemicals and reagents used in this study were of analytical grade. A MilliporeSigma water purification system (Burlington, MA, USA) was used to prepare double-distilled water for LC-MS analysis.

### 3.2. Preparation of Stock and Working Solutions

Primary stock solutions for IWR-1-endo and the ISTD at a concentration of 1 mg/mL were prepared by independent measurements in DMSO and stored in a 4 mL amber vial at −80 °C until further use. The standard IWR-1 solution was diluted in methanol/water (1:1; *v/v*) to obtain calibrant working solutions at 50, 100, 500, 1000, 2500, 5000, 7500, and 10,000 ng/mL for the murine plasma standard curve. For the microdialysate standard curve, calibrant subsolutions were 5, 10, 50, 100, 500, 1000, 2500, and 5000 ng/mL. Similarly, the quality control working solutions for the murine plasma method was 150 ng/mL (low-quality control; LQC), 3000 ng/mL (middle-quality control; MQC), and 8500 ng/mL (high-quality control; HQC). For the microdialysate method, the quality control subsolutions were 15 ng/mL (LQC), 1500 ng/mL (MQC), and 4000 ng/mL (HQC). The ISTD working solution (1 µg/mL) was prepared fresh at the time of assay by serial dilution of the ISTD primary stock solution in methanol/water (1:1; *v/v*).

### 3.3. Calibration Standards and Quality Controls

For the murine plasma curve, the calibration standards were prepared by spiking 2 μL of calibrator working solutions into 20 μL aliquots of blank CD1 murine plasma to yield the following IWR-1 concentrations: 5, 10, 50, 100, 250, 500, 750, and 1000 ng/mL. Similarly, quality control (QC) samples were prepared from respective QC working solutions in blank CD1 murine plasma at IWR-1-endo concentrations of 15 ng/mL (LQC), 300 ng/mL (MQC), and 850 ng/mL (HQC).

Blank microdialysate used for validation studies was made up of Ringer’s solution modified with 10% BCD, which was similar to the perfusate used for the cerebral microdialysis study. For the microdialysis curve, the calibrator standards were prepared by spiking 2 μL of calibrator working solutions into 20 μL aliquots of blank microdialysate to yield IWR-1-endo concentrations of 0.5,1, 5, 25, 50, 125, 250, and 500 ng/mL. Likewise, the quality control samples were prepared from respective QC working solutions in blank microdialysates at IWR-1-endo concentrations of 1.5 ng/mL (LQC), 100 ng/mL (MQC), and 400 ng/mL (HQC).

### 3.4. Plasma and Microdialysate Sample Preparation

Plasma and microdialysate samples were pretreated by solid-phase extraction (SPE) with a Waters^®^ Oasis HLB μElution 96-well plate, 2 mg sorbent per well, 30 μm (Milford, MA, USA). The prepared calibrator standards and QC samples in plasma or microdialysate were spiked with 10 µL of ISTD working solution (1 µg/mL). Double-blank samples (i.e., no analyte or ISTD) were prepared by adding 12 µL of methanol/water (1:1, *v/v*) for volume correction to 20 µL of blank CD1 plasma or microdialysate. Similarly, for blank samples (i.e., no analyte), 20 µL of blank CD1 plasma or microdialysates was spiked with 10 µL of ISTD working solution (1 µg/mL) and 2 μL of methanol/water (1:1, *v/v*) for volume correction. Frozen plasma and microdialysis study samples were initially thawed on ice and vortexed for 30 s. Then, 20 µL aliquots of these study plasma or microdialysate samples were mixed with 10 µL of ISTD working solution (1 µg/mL) and 2 μL of methanol/water (1:1, *v/v*) as volume correction to account for drug spiking in standard and QC samples. The prepared calibrators, QCs, and study sample mixtures were adjusted to a pH of 1.5 with 200 µL dilute hydrochloric acid (0.1 N). Next, acidified sample mixtures were vortex mixed for 1 min, centrifuged at 12,000× *g* for 5 min at 4 °C and applied to an HLB μElution plate that was earlier conditioned with 2 × 100 μL of methanol and equilibrated with 2 × 100 μL of distilled water. The loaded plasma or microdialysate samples were allowed to pass through the plate wells with minimum positive pressure. Wells were rapidly flushed with 2 × 200 μL of distilled water. Then, maximum positive pressure was applied to completely dry the plate wells, followed by elution of the analyte with 2 × 70 μL of acetonitrile: methanol solution mixture (9:1, *v/v*) into a clean 96-well collection plate (500 µL round-bottom well plate) from Wheaton (Millville, NJ, USA). The plate was sealed using a Wheaton^®^ silicone cap mat (Millville, NJ, USA) after adding distilled water (60 μL) to all sample wells. Lastly, the contents of this plate were vortex mixed for 30 s, centrifuged at 4000× *g* for 2 min at 4 °C, and the plate was placed in the autosampler rack. Volumes of 2 µL and 10 μL of the final sample extracts were injected onto the C_18_ column for LC-MS/MS analysis of murine plasma and microdialysate samples, respectively.

### 3.5. Chromatographic Conditions

Liquid chromatography was performed using a Shimadzu Nexera X2 high-performance liquid chromatograph (Kyoto, Japan) equipped with a binary pump (LC-30AD), a degasser (DGU-A_5_), an autosampler (SIL-30AC), a controller (CBM-20A), and a column oven (Thermasphere TS-130). A Kinetex^®^ HPLC C_18_ column (100A, 50 × 2.1 mm, 4 µm particle size) attached to a KrudKatcher^TM^ Ultra HPLC In-Line Filter (2 µm Depth Filter × 0.004 in ID; Phenomenex, Torrance, CA, USA) was used for chromatographic separation of IWR-1-endo and ISTD. The mobile phase for the separation of analytes included solution A (distilled water with 0.1% formic acid) and B (acetonitrile with 0.1% formic acid). The following gradient profile was used: 0–0.2 min, 5–50% B; 0.2–0.8 min, 50% B; 0.8–0.9 min, 50–75% B; 0.9–2.2 min, 75% B; 2.2–2.3 min, 75–98% B; 2.3–3.1 min, 98% B; 3.1–3.25 min, 98–5% B; 3.25–5.0 min, 5% B. This gradient phase was pumped at a constant flow rate of 0.6 mL/min throughout the sample run. The total LC-MS/MS run time per sample was 5 min. The injection volumes into the LC-MS/MS for determining IWR-1-endo from murine plasma and microdialysates were 2 µL and 10 µL per sample, respectively. The column temperature and autosampler temperature were kept at 45 ± 1 °C and 4 ± 1 °C, respectively, throughout all measurements. During the sample batch run, the autosampler needle and port were rinsed using a strong flushing solution of methanol/acetonitrile/water/2-propanol (1:1:1:1; *v/v*) modified with 0.1% formic acid.

### 3.6. Mass Spectrometry Conditions

IWR-1-endo and dabrafenib (ISTD) detection and quantification were performed using the Applied Biosystems Hybrid Q-Trap 4000 mass spectrometer (Framingham, MA, USA) equipped with the Turbo Ion Spray probe. The instrument control and data processing software Sciex Analyst^®^ (version 1.7.1) was used for spectral data acquisition, peak integration, and quantification. Mass detection was performed in the positive electrospray ionization mode and the mass transitions monitored were 410.40 > 344.10 (IWR-1-endo; Figure 2A) and 520.40 > 307.20 (ISTD; Figure 2B). Mass resolutions were set at 0.7 full width at half height (unit resolution) for both Q1 and Q3 quadrupoles. The compound-dependent parameters such as declustering potential (DP), entrance potential (EP), collision energy (CE), and collision exit potential (CXP) were set at 105.0 V, 11.0 V, 25.0 eV, and 10.0 V, respectively, for IWR-1-endo; for ISTD, it was 125.0 V, 9.0 V, 30.0 eV, and 8.0 V, respectively. The compound independent parameters including curtain gas (CUR), ion spray voltage (ISP), nebulizer gas (GS1), and heater gas (GS2) were set at 30 psi, 5500 V, 50 psi, and 50 psi, respectively, for both IWR-1-endo and ISTD. The ion source temperature was maintained at 600 °C, and the dwell time for monitoring both the transitions was set to 200 ms.

### 3.7. Method Validation Procedures

Using the FDA Guidance for Industry, Bioanalytical Method Validation, May 2018, this LC-MS/MS method was validated in terms of linearity, selectivity, sensitivity (lower limit of quantitation, limit of detection), precision, accuracy, matrix effects, recovery, stability, carry-over, and dilution integrity [16].

#### 3.7.1. Linearity

The calibration curve was constructed by analyzing a double blank, a blank, and eight nonzero calibrator standards in blank murine CD1 plasma and Ringer’s/BCD solution on three successive days. Standard curves were plotted using peak area ratios of analyte to ISTD (y-axis) against the nominal analyte concentrations (x-axis). A linear regression equation, with a weighing factor of 1/x^2^, was used to get the best fit for the IWR-1-endo concentration/peak area ratio relationship. The linear ranges tested for the murine plasma and microdialysate methods were 5–1000 ng/mL and 0.5–500 ng/mL, respectively. The calibration curves were considered linear/acceptable when at least 75% of the calibration standards, including the LLOQ and ULOQ, had accuracy values for the measured concentrations within a ±15% range, except ±20% for the LLOQ, and the correlation coefficient (r^2^) was ≥0.99.

#### 3.7.2. Selectivity and Sensitivity

Selectivity studies were performed to evaluate the interference in analyte estimation due to the presence of endogenous substances in murine plasma or microdialysates. Selectivity studies were conducted in two different mice plasma samples viz. CD1 mice and CD1 nude mice and Ringer’s/BCD solution, with a total of six replicates in each matrix at double blank (no analyte or ISTD spiked) and LLOQ level. The acceptance criteria for demonstrating selectivity were that double-blank plasma or microdialysates should not exhibit interfering peak responses at the retention time of IWR-1-endo ≥20% of the LLOQ peak response and ≥5% ISTD peak response in the same matrix.

The sensitivity of the developed method was calculated in terms of the analyte signal-to-noise (S/N) ratio. The LOD was determined using blank CD-1 murine plasma or Ringer’s/BCD solution (n = 6) spiked before extraction to concentrations that were estimated to give S/N ratios ≥3 based on initial method development data. The LLOQ was defined as having a signal-to-noise ratio (S/N) ≥10 with bias and coefficient of variation (CV) ≤20% by analyzing six replicates of blank CD1 murine plasma or Ringer’s/BCD solution.

#### 3.7.3. Precision and Accuracy

The within-run precision and accuracy (P & A) were determined by the QC samples at four concentration levels (LLOQ, LQC, MQC, and HQC) distributed across the calibration curve in six replicates during the same day. The between-run precision and accuracy were ascertained by assaying the QC samples on three independent days in six replicates. Precision and accuracy were expressed in terms of relative standard deviation (RSD) and relative error (RE), respectively. The acceptance criteria for within-run (n = 6) and between-run (n = 18) accuracy was that the REs obtained for each QC level were within ±15% of the nominal concentrations except for the LLOQ level, where ± 20% were acceptable. The acceptance criteria for within-run (n = 6) and between-run (n = 18) precision were that the RSDs obtained for each QC level were ≤15% except for the LLOQ level, where ≤20% were acceptable. Further, for acceptance of a single P & A batch run, a minimum of 50% of QC samples and 2/3rd (67%) of the total QC samples assessed had to meet the acceptance criteria.

#### 3.7.4. Matrix Effect and Recovery

The samples for estimating the matrix effect for IWR-1-endo and ISTD were prepared in blank microdialysate, and blank murine plasma obtained from two mice strains (CD-1 and CD-1 nude mice). The matrix effect was calculated as a matrix factor, which was the ratio of the instrument’s response for post-extracted spiked samples to that of the instrument response for neat standard solutions at equivalent concentrations. For preparing post-extracted spiked samples, blank microdialysate or plasma samples were prepared as per the sample preparation protocol and later spiked at the QC levels. A value of MF close to 1 indicates no or negligible matrix effect, while MF ≤ 0.8 and ≥1.2 demonstrates ion suppression and ion enhancement, respectively [17,18,19]. Similarly, mean extraction recoveries for IWR-1-endo and ISTD were computed as the ratio of instrument response for the extracted samples to that of the instrument response for the post-extracted spiked samples. The matrix effect and recovery experiments were performed in triplicates at the LQC and HQC concentration levels for both the developed LC-MS/MS methods.

#### 3.7.5. Stability

The stability studies were performed in blank plasma samples and microdialysates at the LQC and HQC concentrations (n = 3 replicates). They were studied under the following conditions: freeze–thaw cycle stability (FTS), short-term stability (STS), long-term stability (LTS), and postprocessing stability. For freeze–thaw cycle stability studies, the plasma or microdialysate samples were frozen at −80 °C for 24 h and then thawed for 1 h at room temperature and frozen again for 24 h, repeating this process until the third thawing cycle. After each freeze–thaw cycle, samples were extracted and analyzed. The STS studies were assessed for the plasma or microdialysate samples kept at ambient temperature and 4 °C for 48 h. The samples were extracted and analyzed over specified intervals to ascertain the STS for IWR-1-endo.

For establishing postprocessing stability in the autosampler, extracted samples were stored in the autoinjector at 4 °C for 48 h and then injected into the LC-MS/MS system. The LTS studies in murine plasma and microdialysate were conducted at –80 °C for 21 and 29 days, respectively. Samples were considered stable if the percentage difference in concentration was within 15% of the freshly processed quality control samples.

#### 3.7.6. Carry-Over

Carry-over was evaluated by sequentially injecting a ULOQ sample (1000 ng/mL for the murine plasma method and 500 ng/mL for the microdialysate method) and immediately followed by the injection of two extracted double-blank samples. The concentration of the ISTD spiked in the ULOQ samples was 500 ng/mL for both the murine plasma and the microdialysate methods. A carry-over was considered negligible if the analyte peak area responses in the blank samples were ≤20% of the LLOQ peak response and ≤5% of the ISTD peak response.

#### 3.7.7. Dilution Integrity

Dilution integrity was performed to test the ability of diluted microdialysis study samples to yield accurate results, specifically applying to study samples whose concentrations are above the upper limit of quantification (ULOQ). To study the dilution effect, murine plasma samples with initial concentrations of 4250 ng/mL and 8000 ng/mL were diluted 5- and 10-fold with blank CD1 plasma, respectively. For the microdialysate method, microdialysate samples with initial concentrations of 2000 ng/mL and 4000 ng/mL were diluted 5- and 10-fold with the blank microdialysate, respectively. Six replicates of diluted samples were processed, analyzed, and compared to a fresh calibration curve. The acceptance criteria for dilution integrity were that the concentrations obtained after applying the dilution factor were within ±15% of the nominal concentrations and % C.V. not more than 15% within replicates.

### 3.8. Method Application to Cerebral Microdialysis Study

A cerebral microdialysis study was performed to sample brain extracellular fluid (ECF) from a non-tumor-bearing female CD1 nude mouse (Charles River, Wilmington, MA) as per a procedure previously described and approved by the St. Jude Institutional Animal Care and Use Committee [20]. A microdialysis probe with a 38 kDa MWCO semi-permeable membrane (MD-2211, BASi) was inserted into a guide cannula implanted in the cerebral cortex of the animal. The microdialysis probe was perfused with Ringer’s solution containing 10% BCD at a flow rate of 0.5 μL/min and equilibrated for 1 h. BCD was added to the perfusate to reduce the non-specific binding of IWR-1-endo to the microdialysis system. Then, the mouse was dosed with 30 mg/kg IWR-1-endo via intraperitoneal injection. Dosing formulation (3 mg/mL) comprised 7.5% NMP, 7.5% Solutol HS-15, 30% PEG-400, and 55% TPGS (10% *w/v* solution) and was vortexed for 2 min to obtain a clear solution. After dosing, brain dialysate fractions were collected over 1 h intervals for a total of 6 h. Blood samples were collected retro-orbitally at 0.25, 2.5, and 6 h after dosing and spun down to plasma within 2 min of collection. All plasma and dialysate samples (20 µL) were immediately transferred into siliconized tubes containing 200 µL of 0.1 N HCl and stored at −80 °C. Microdialysis probe recovery was assessed to calculate brain ECF concentrations using the zero-flow rate method, with dialysates collected at 0.5, 1, 1.5, and 2 µL/min, as previously described [21].

## 4. Conclusions

In this study, we developed and validated a rapid, simple, and sensitive LC-MS/MS method for the measurement of IWR-1-endo in murine plasma and microdialysate samples. Major highlights of the LC-MS/MS method include the ability to quantify IWR-1-endo over a wide dynamic range (5–1000 ng/mL for the murine plasma curve and 0.5–500 ng/mL for the microdialysate curve) in a relatively small sample volume (20 µL), coupled with a short analysis time of 5 min. To summarize, our study shows that IWR-1-endo is relatively stable in working solutions; however, the drug rapidly degrades in murine plasma and brain microdialysate samples. Furthermore, adjusting the pH of the samples with dilute hydrochloric acid to 1.5 dramatically improved the stability of IWR-1-endo in murine plasma and brain microdialysate samples. Lastly, the validated LC-MS/MS methods in murine plasma and brain microdialysates were successfully applied to cerebral microdialysis studies in CD-1 nude mice after the administration of IWR-1-endo. The findings of this study could serve as a valuable reference for future development and clinical applications of IWR-1-endo.

## Figures and Tables

**Table 1:** Parameters for calibration curves in different matrices.

Parameter	Sample Matrix	
	Murine Plasma	Microdialysates
Calibration Range (ng/mL)	5–1000	0.5–500
LLOQ (ng/mL)	5	0.5
LOD (ng/mL)	0.5	0.2
Intercept (a)	−1.50 × 10^−3^	1.70 × 10^−4^
Sa	1.20 × 10^−3^	1.17 × 10^−4^
Slope (b)	3.90 × 10^−3^	1.33 × 10^−2^
Sb	0.20 × 10^−3^	0.07 × 10^−2^
Correlation Coefficient (r^2^)	0.9989	0.9990

Sa: standard deviation of intercept; Sb: standard deviation of the slope; r^2^: coefficient of determination; LOD: limit of detection; LLOQ, lower limit of quantification.

**Table 2:** Within-run and between-run precision and accuracy for IWR-1-endo in murine plasma (n = 6 for within-run and n = 18 for between-runs).

P & A Batch Run	Replicates	Murine Plasma			
		Nominal Conc.	Calculated conc.	Accuracy	Precision
	(n)	(ng/mL)	(ng/mL)	(% R.E.)	(% R. S. D.)
Within-Run 1	6	5	5.30	6.07	5.04
	6	15	15.06	0.38	4.06
	6	300	312.81	4.27	2.89
	6	850	895.12	5.31	1.74
Within-Run 2	6	5	5.09	1.75	10.10
	6	15	14.87	−0.86	5.87
	6	300	306.98	2.33	2.29
	6	850	908.24	6.85	6.80
Within-Run 3	6	5	5.46	9.26	6.58
	6	15	15.65	4.31	4.50
	6	300	324.06	8.02	3.30
	6	850	924.98	8.82	1.03
Average of Runs 1, 2, and 3 (Between-Runs)	18	5	5.28	5.69	7.61
	18	15	15.19	1.28	5.08
	18	300	314.62	4.87	3.56
	18	850	909.45	6.99	4.08

% R.E.: percent relative error; % R.S.D.: percent relative standard deviation.

**Table 3:** Within-run and between-run precision and accuracy for IWR-1-endo in microdialysates (n = 6 for within-run and n = 18 for between-runs).

P & A Batch Run	Replicates	Microdialysate			
		Nominal Conc.	Calculated conc.	Accuracy	Precision
	(n)	(ng/mL)	(ng/mL)	(% R.E.)	(% R. S. D.)
Within-Run 1	6	0.5	0.46	−8.86	3.33
	6	1.5	1.39	−7.25	5.25
	6	150	141.88	−5.42	2.25
	6	400	372.08	−6.98	4.66
Within-Run 2	6	0.5	0.49	−1.16	6.95
	6	1.5	1.49	−0.53	7.31
	6	150	148.13	−1.24	4.11
	6	400	399.15	−0.21	3.29
Within-Run 3	6	0.5	0.49	−2.36	3.68
	6	1.5	1.44	−3.69	6.11
	6	150	144.89	−3.41	3.29
	6	400	395.57	−1.11	3.32
Average of Runs 1, 2, and 3 (Between-Runs)	18	0.5	0.48	−4.13	5.95
	18	1.5	1.44	−3.82	6.64
	18	150	144.97	−3.36	3.62
	18	400	388.94	−2.77	4.76

% R.E.: percent relative error; % R.S.D.: percent relative standard deviation.

**Table 4:** Matrix factors and recovery in various matrices (n = 3).

Sample Matrix	Compound	Nominal Concentration (ng/mL)	Matrix Effect		Recovery	
			Mean Calculated MF value	% R.S.D.	% Mean Recovery	% R.S.D.
Mouse Plasma CD1	IWR-1	15	1.02	2.55	97.16	1.57
		850	1.00	1.07	97.92	1.94
	ISTD	500	0.97	1.07	102.45	1.52
Mouse plasma CD1 Nude	IWR-1	15	0.99	2.51	100.67	2.40
		850	1.00	1.81	97.34	3.23
	ISTD	500	0.95	0.64	104.58	2.24
Microdialysate	IWR-1	1.5	1.05	9.73	86.56	10.00
		150	1.13	1.64	90.96	3.85
	ISTD	500	0.95	3.85	94.94	4.78

**Table 5:** Summary of stability evaluation for IWR-1-endo in murine plasma and microdialysates (n = 3).

Sample Matrix	Stability Study	Nominal Concentration (ng/mL)	Mean ± S.D. Calculated Concentration (ng/mL)	Precision (% R.S.D.)	Accuracy (% R.E.)	% Mean Deviation
Murine Plasma	Process ^a^	15	16.33 ± 0.63	3.87	8.88	9.35
		850	916.82 ± 10.54	1.15	7.86	12.40
	Bench-Top RT ^b^	15	15.87 ± 0.45	2.84	5.82	6.27
		850	915.08 ± 14.18	1.55	7.66	12.18
	Bench-Top 4 °C ^c^	15	15.20 ± 0.24	1.55	1.34	1.78
		850	915.69 ± 16.12	1.76	7.73	12.26
	Freeze–Thaw ^d^	15	16.01 ± 0.88	5.48	6.70	7.16
		850	917.18 ± 30.24	3.30	7.90	12.44
	Long-Term ^e^	15	15.93 ± 0.21	1.35	6.17	−5.65
		850	938.89 ± 15.84	1.77	10.46	−0.33
Microdialysate	Process ^a^	1.5	1.56 ± 0.042	2.68	4.16	7.79
		400	429.35 ± 7.00	1.63	7.34	6.64
	Bench-Top RT ^b^	1.5	1.60 ± 0.05	3.02	6.35	9.35
		400	434.08 ± 17.01	3.92	8.52	7.61
	Bench-Top 4 °C ^c^	1.5	1.60 ± 0.08	4.86	6.40	11.50
		400	415.84 ± 6.85	1.65	3.96	3.09
	Freeze–Thaw ^d^	1.5	1.54 ± 0.07	4.56	2.34	5.91
		400	424.79 ± 9.18	2.16	6.20	5.31
	Long-Term ^f^	1.5	1.39 ± 0.008	0.60	−7.63	−4.41
		400	390.35 ± 21.53	5.52	−2.41	−3.23

%R.S.D.: relative standard deviation; %R.E.: relative error; ^a^ Stability assessed after 48 h in autosampler at 4 °C; ^b^ Short-term stability after 48 h at room temperature; ^c^ Short-term stability after 48 h at 4 °C; ^d^ Stability evaluated after three freeze–thaw cycles; ^e^ 21 days long-term stability in murine plasma at −80 °C; ^f^ 29 days long-term stability in microdialysate at −80 °C.

## Supplementary Materials

The following supporting information can be downloaded at: https://www.mdpi.com/article/10.3390/molecules27175448/s1, Figure S1: Representative extracted-ion chromatograms for IWR-1-endo (1 ppm prepared in methanol/water, 1:1 *v/v*) in (A) acetonitrile/water system modified with 0.1 % formic acid and (B) methanol/water system modified with 0.1% formic acid. A gradient profile was employed for both the optimization, as described in Section 3.5; Figure S2: Representative extracted-ion chromatograms for IWR-1-endo and ISTD (1 ppm prepared in methanol/water, 1:1 *v/v*) on (A) Kinetex^®^ C_8_ analytical column (100°A, 50 × 2.1 mm, 2.6 µm particle size) and (B) Kinetex^®^ C_18_ analytical column (100°A, 50 × 2.1 mm, 2.6 µm particle size). A gradient profile with acetonitrile/water system (0.1 % formic acid added) was employed for both the optimization, as described in Section 3.5; Figure S3: Stability evaluation of IWR-1 in murine plasma stored at 4 °C and room temperature over a period of 24 h (n = 3; mean ± S.D.) with enzyme inhibitors (A) p-chloromercuribenzoate, (B) bis-(p-nitrophenyl) phosphate, and (C) sodium fluoride. LQC: low-quality control; HQC: high-quality control; RT: room temperature; Figure S4: Stability assessment of IWR-1 in murine plasma stored at 4 °C and room temperature over a period of 24 h (n = 3; Mean ± S.D.) under different pH conditions (A) LQC level at RT, (B) HQC level at RT, (C) LQC level at 4 °C, and (D) HQC level at 4 °C. Plasma pH adjustments of 1.5, 7.0, and 11.0 were performed using 0.1 N hydrochloric acid, 1M sodium phosphate buffer, and 0.1 N sodium hydroxide, respectively. LQC: low-quality control; HQC: high-quality control; RT: room temperature.; Figure S5: Representative calibration curves for IWR-1-Endo in (A) mouse plasma and (B) microdialysates. Both curves were fitted to calibrators using weighted 1/x^2^ linear regression; Table S1: Optimization of IWR-1-endo recovery from mouse plasma and microdialysates using SPE µElution plates (n = 3).
