# Supplementary material for: Development and Validation of a Sensitive and Specific LC-MS/MS Method for IWR-1-Endo, a Wnt Signaling Inhibitor: Application to a Cerebral Microdialysis Study
Source: Molecules. 2022 Aug 25;27(17):5448. doi: 10.3390/molecules27175448 (PMC9457781; doi:10.3390/molecules27175448)
Supplement: Supplementary file 1 [file molecules-27-05448-s001.zip › molecules-1850791-SI.pdf]

molecules

Article

Development and Validation of a Sensitive and Specific LC-MS/MS Method for IWR-1-Endo, a Wnt Signaling Inhibitor: Application to a Cerebral Microdialysis Study

Sreenath Nair , Abigail Davis, Olivia Campagne, John D. Schuetz and Clinton F. Stewart *

Department of Pharmacy and Pharmaceutical Science, St. Jude Children's Research Hospital,

Memphis, TN 38105, USA

* Correspondence: clinton.stewart@stjude.org; Tel.: +1-(901)-595-3665; Fax: +1-(901)-595-3125

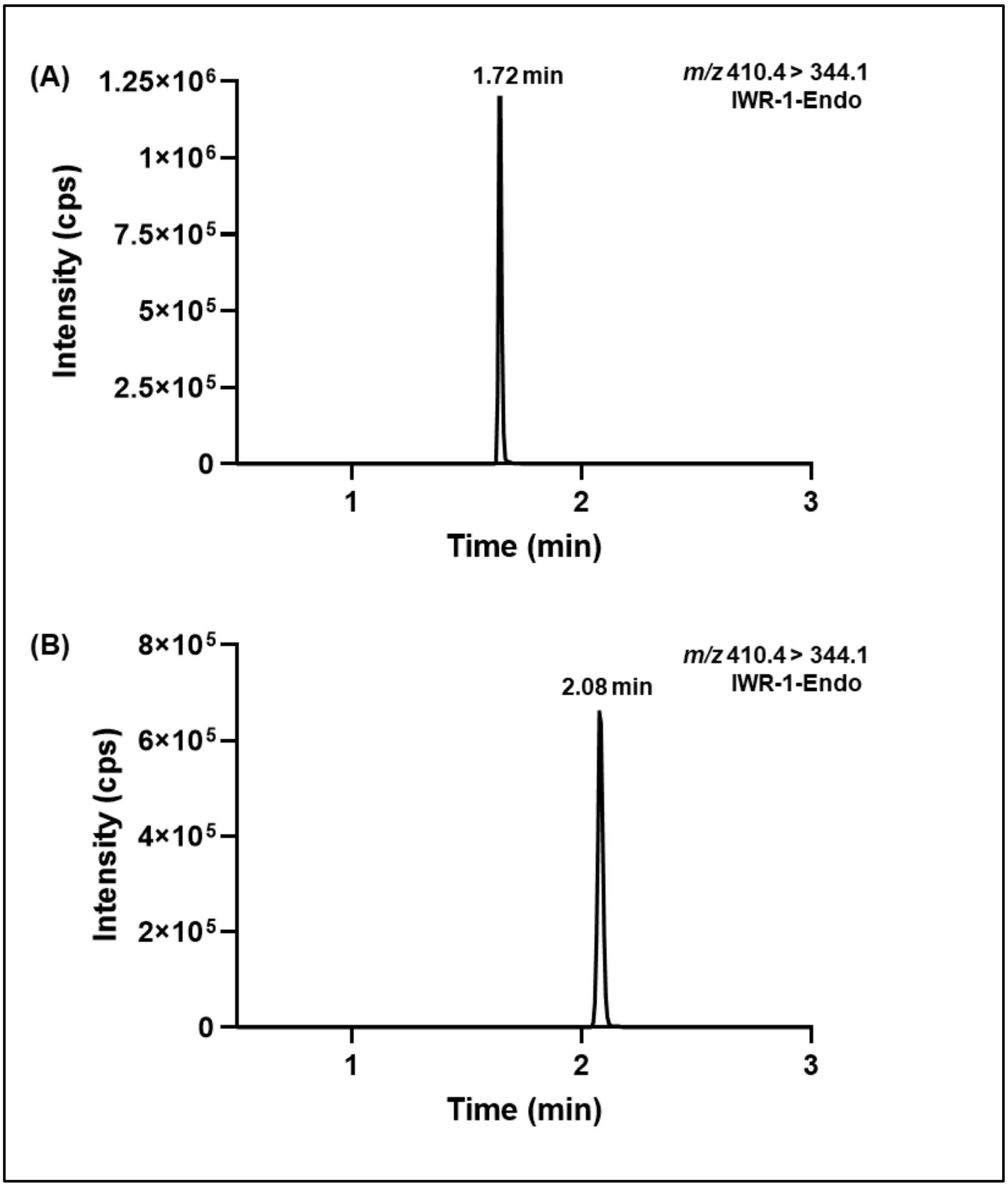

Figure S1:: Representative extracted ion chromatograms for IWR-1-Endo (1 ppm prepared in Methanol: water, 1:1 *v*/*v*) in (A) acetonitrile-water system modified with 0.1 % formic acid and (B) Methanol-water system modified with 0.1% formic acid. A gradient profile was employed for both the optimization as described in section 3.5.

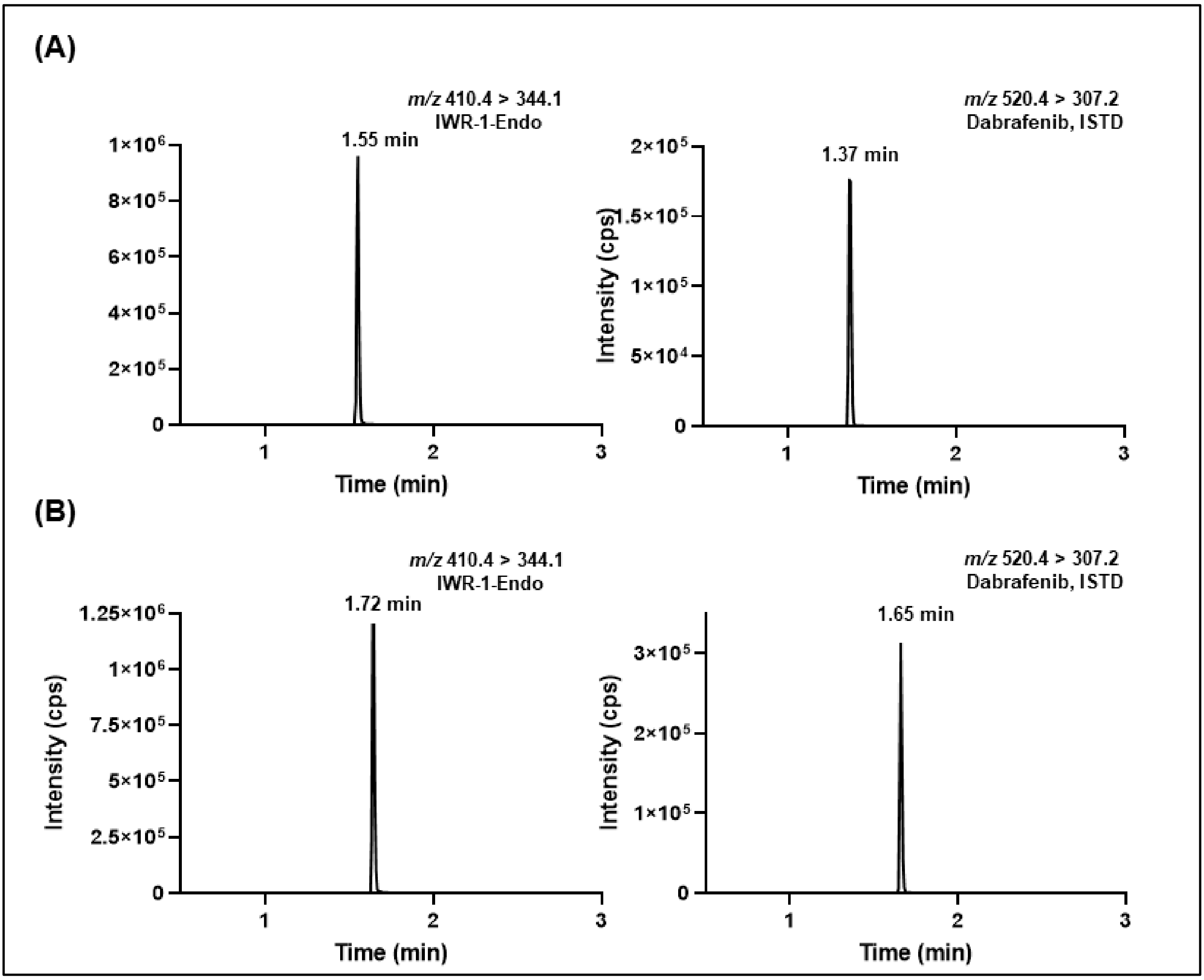

Figure S2: Representative extracted ion chromatograms for IWR-1-endo and ISTD (1 ppm combination standard prepared in Methanol: water, 1:1 *v*/*v*) on (A) Kinetex® C_8 analytical column (100°A, 50 × 2.1 mm, 2.6 µm particle size) and (B) Kinetex® C_{18} analytical column (100°A, 50 × 2.1 mm, 2.6 µm particle size. A gradient profile with acetonitrile-water system (0.1 % formic acid added) was employed for both the optimization as described in section 3.5.

Table S1: Optimization of IWR-1-endo recovery from mouse plasma and microdialysates using SPE µElution plates (n=3)

SPE Method development µElution plate type	Recovery					
	Mouse Plasma CD1			Microdialysate		
	IWR-1-Endo Nominal Concentration (ng/mL)	% Mean Recovery	% R.S.D.	IWR-1-Endo Nominal Concentration (ng/mL)	% Mean Recovery	% R.S.D.
SPE with HLB	15	92.06	3.24	1.50	84.01	5.01
SPE with MAX	15	78.81	2.44	1.50	71.24	4.99
SPE with MCX	15	73.21	10.13	1.50	69.88	10.42
SPE with WAX	15	55.33	6.90	1.50	57.83	9.05
SPE with WCX	15	50.75	13.91	1.50	48.10	12.60

SPE: Solid Phase extraction; HLB: hydrophilic-lipophilic-balanced; MAX: mixed-mode anion exchange; MCX: mixed-mode cation exchange; WAX: mixed-mode weak anion exchange; WCX: mixed-mode weak cation exchange

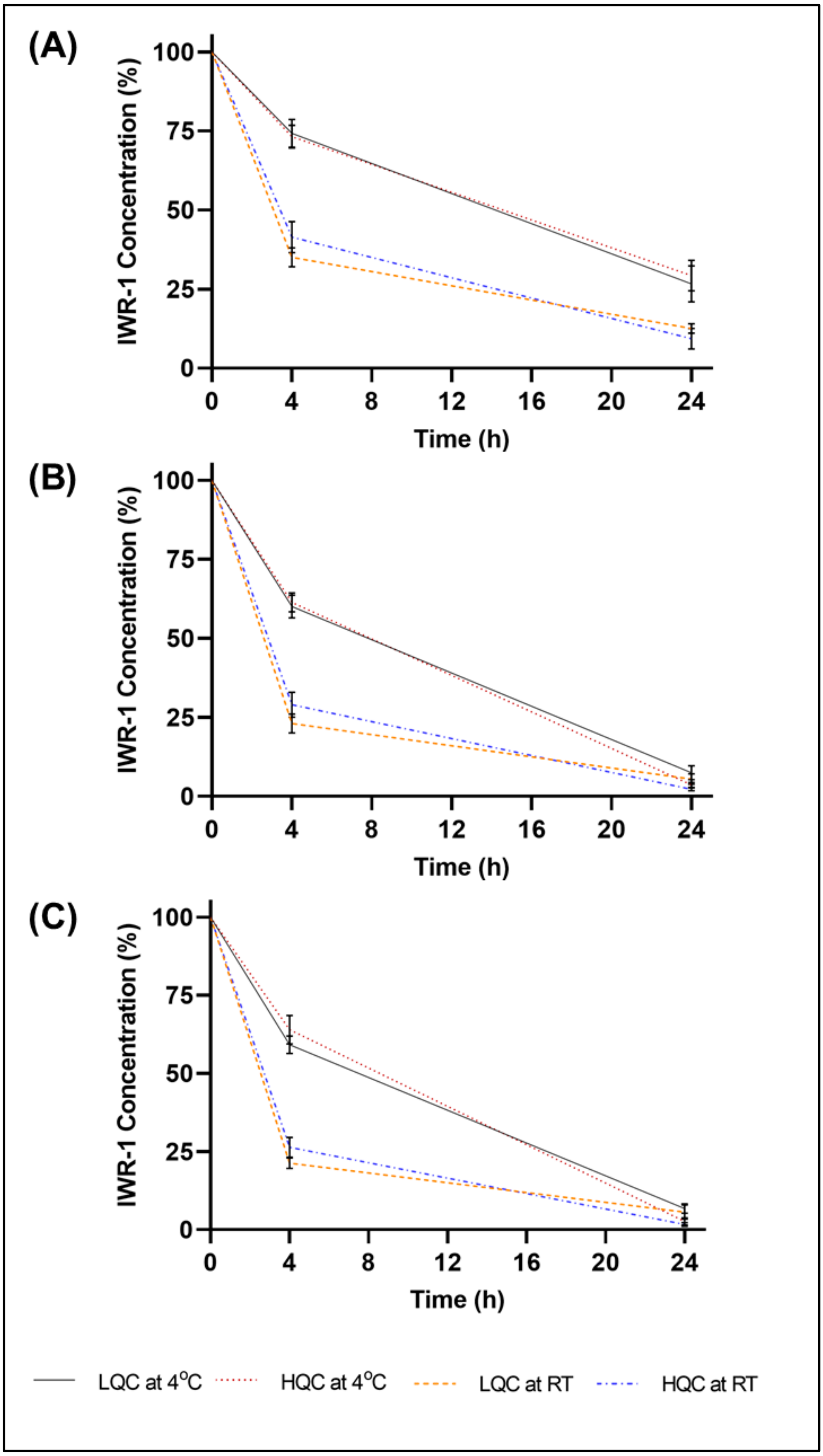

Figure S3. Stability evaluation of IWR-1 in murine plasma stored at 4 °C and room temperature over a period of 24 h (n=3; Mean ± S.D.) with enzyme inhibitors (A) p-chloromercurobenzoate (B) bis-(p-nitrophenyl) phosphate (C) sodium fluoride. LQC: Low quality control; HQC: High quality control; RT: Room Temperature

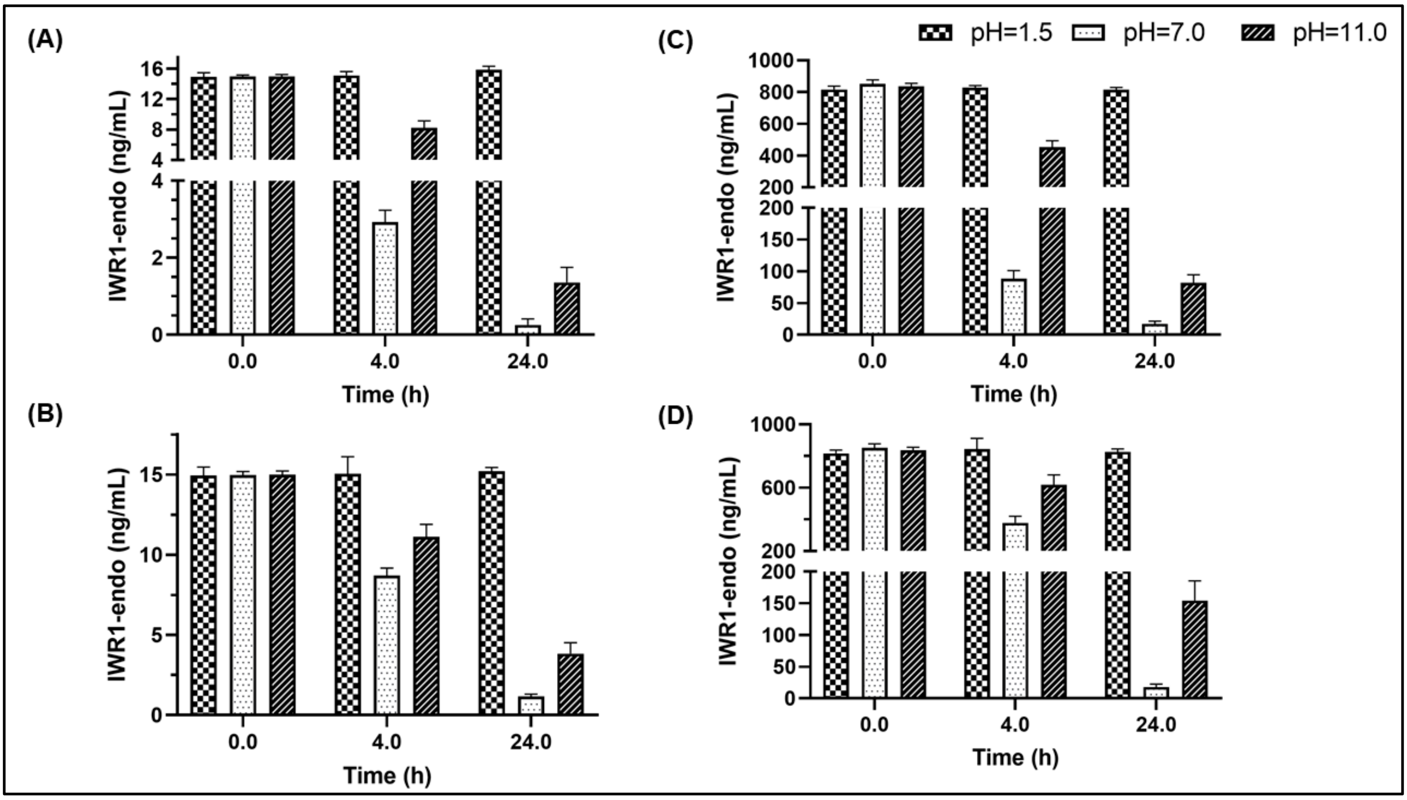

Figure S4. Stability assessment of IWR-1 in murine plasma stored at 4 °C and room temperature over a period of 24 h (n=3; Mean ± S.D.) under different pH conditions (A) LQC level at RT, (B) HQC level at RT, (C) LQC level at 4 °C, and (D) HQC level at 4 °C. Plasma pH adjustments of 1.5, 7.0, and 11.0 were performed using 0.1N hydrochloric acid, 1M sodium phosphate buffer, and 0.1 N sodium hydroxide, respectively. LQC: Low quality control; HQC: High quality control; RT: Room Temperature.

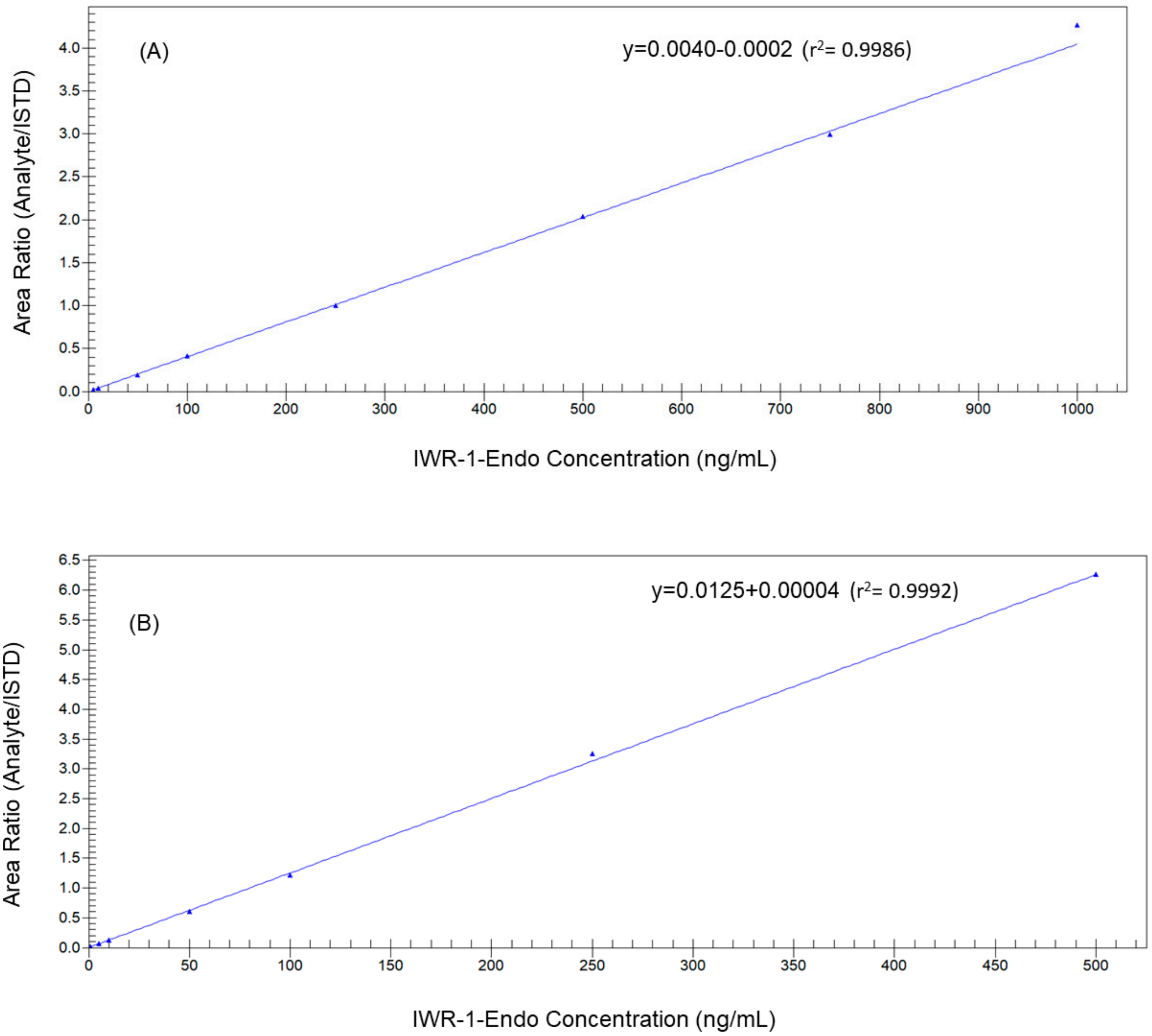

Figure S5: Representative calibration curves for IWR-1-Endo in (A) Mouse plasma and (B) Microdialysates. Both curves were fitted to calibrators using weighted $1/x^2$ linear regression. ISTD: Internal Standard.
