# Supplementary figures and images for: Development and Validation of a Sensitive and Specific LC-MS/MS Method for IWR-1-Endo, a Wnt Signaling Inhibitor: Application to a Cerebral Microdialysis Study
Source: Molecules. 2022 Aug 25;27(17):5448. doi: 10.3390/molecules27175448 (PMC9457781; doi:10.3390/molecules27175448)

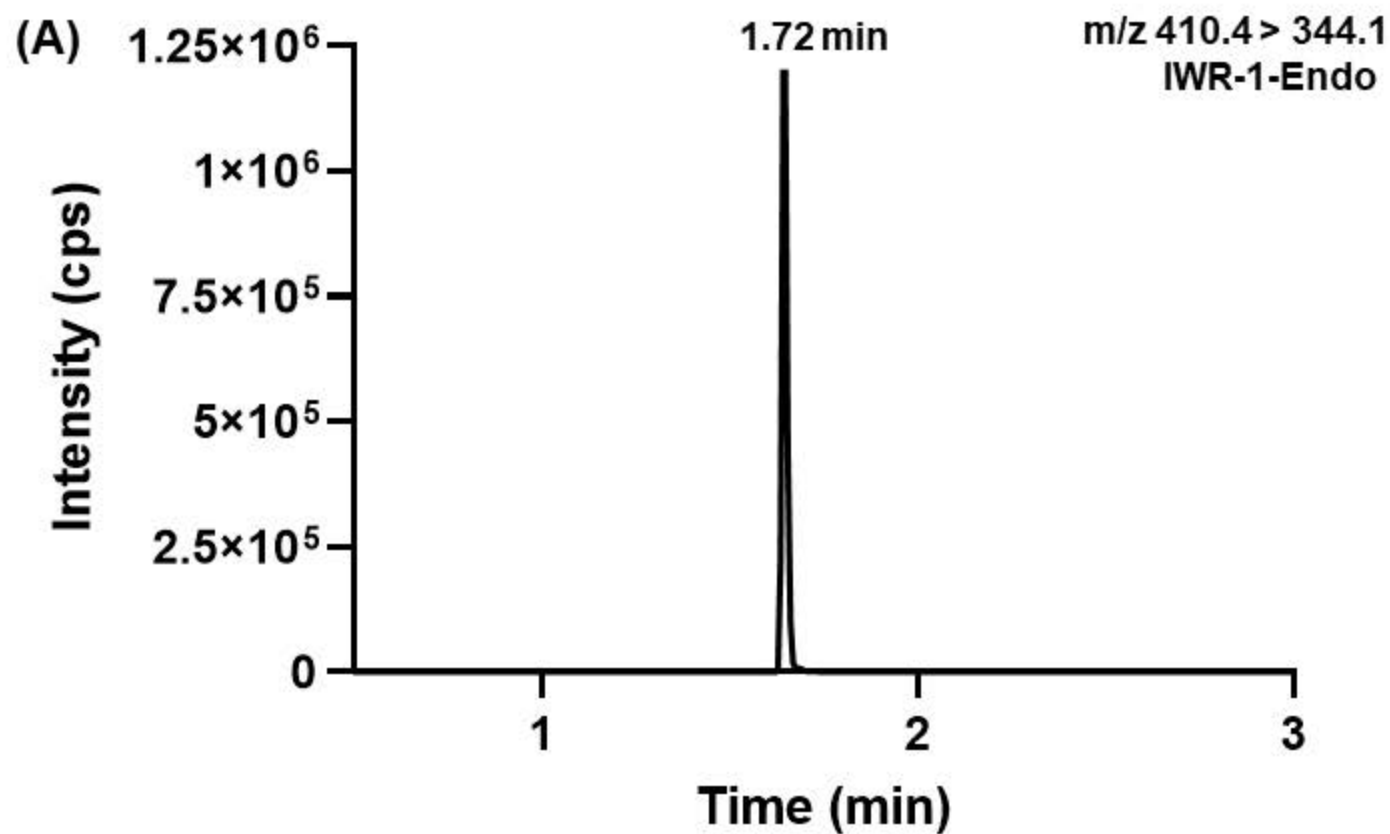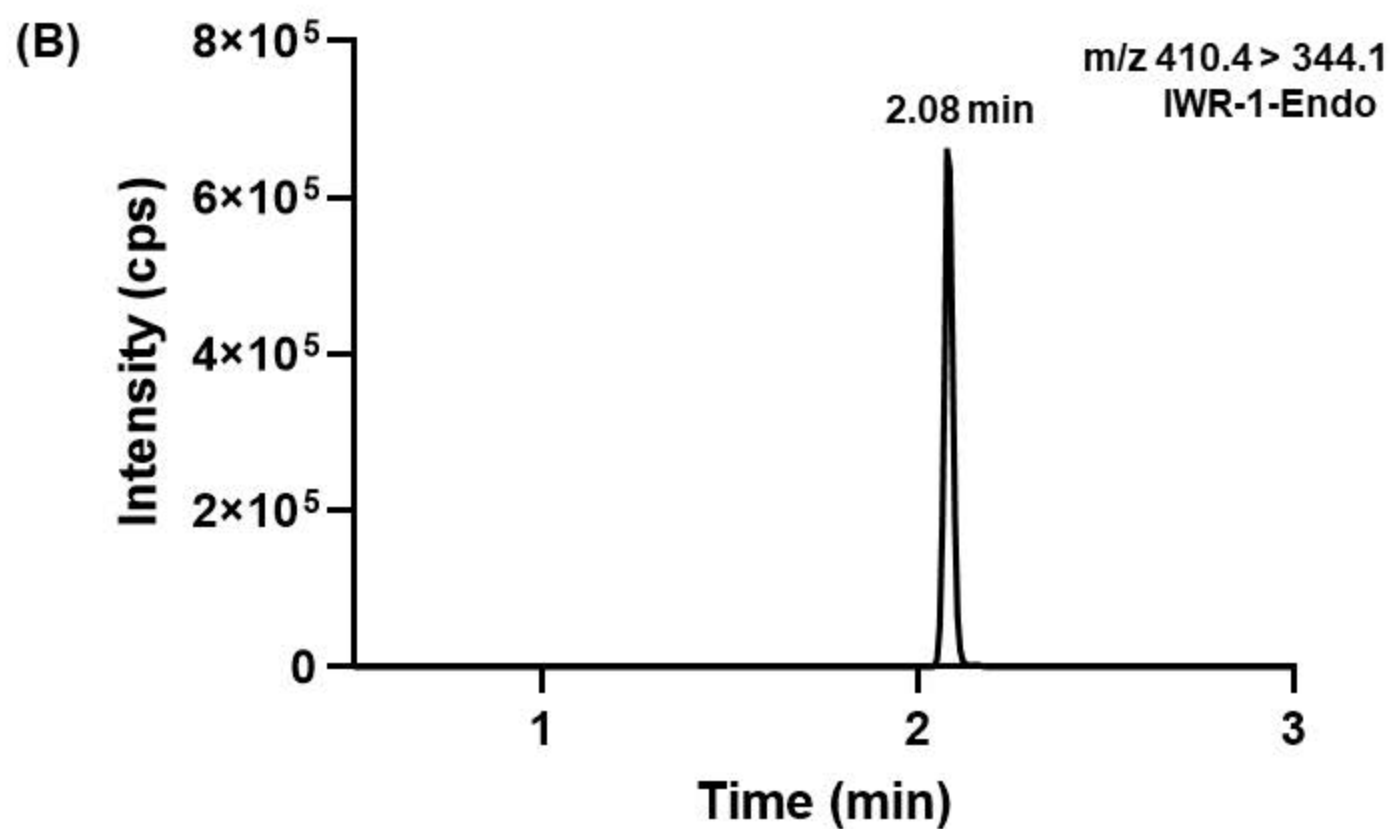

Supplement: Supplementary file 1 [file molecules-27-05448-s001.zip › Supplementary Figure S1.pdf]

(A)

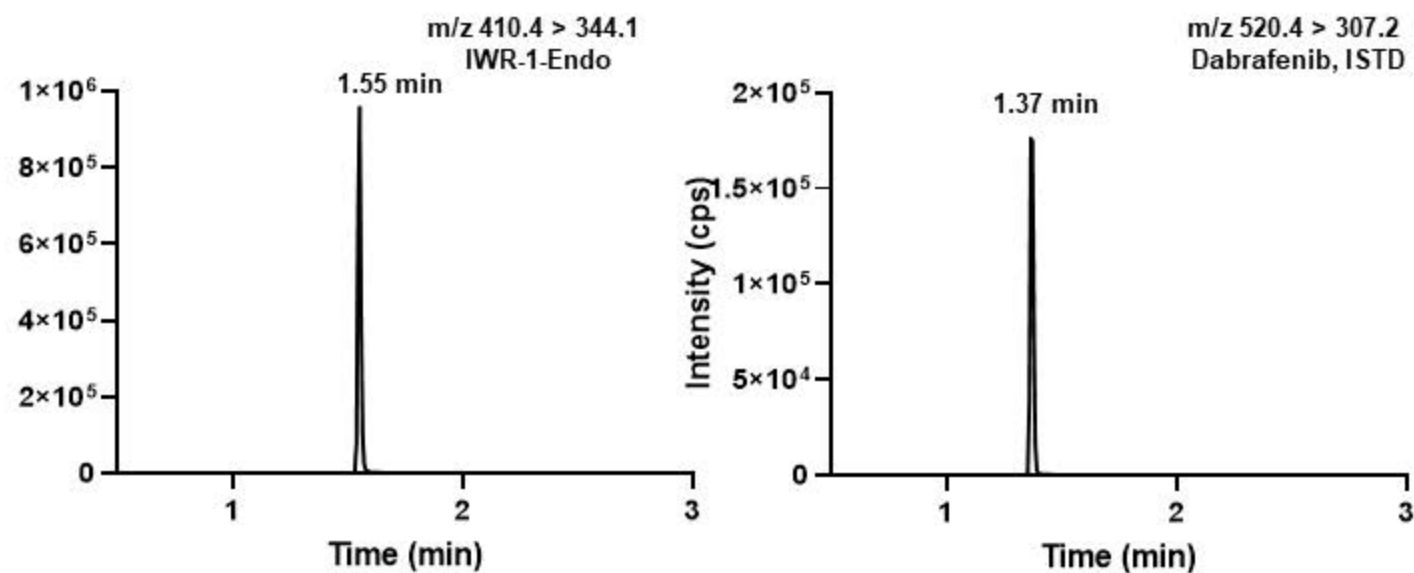

(B)

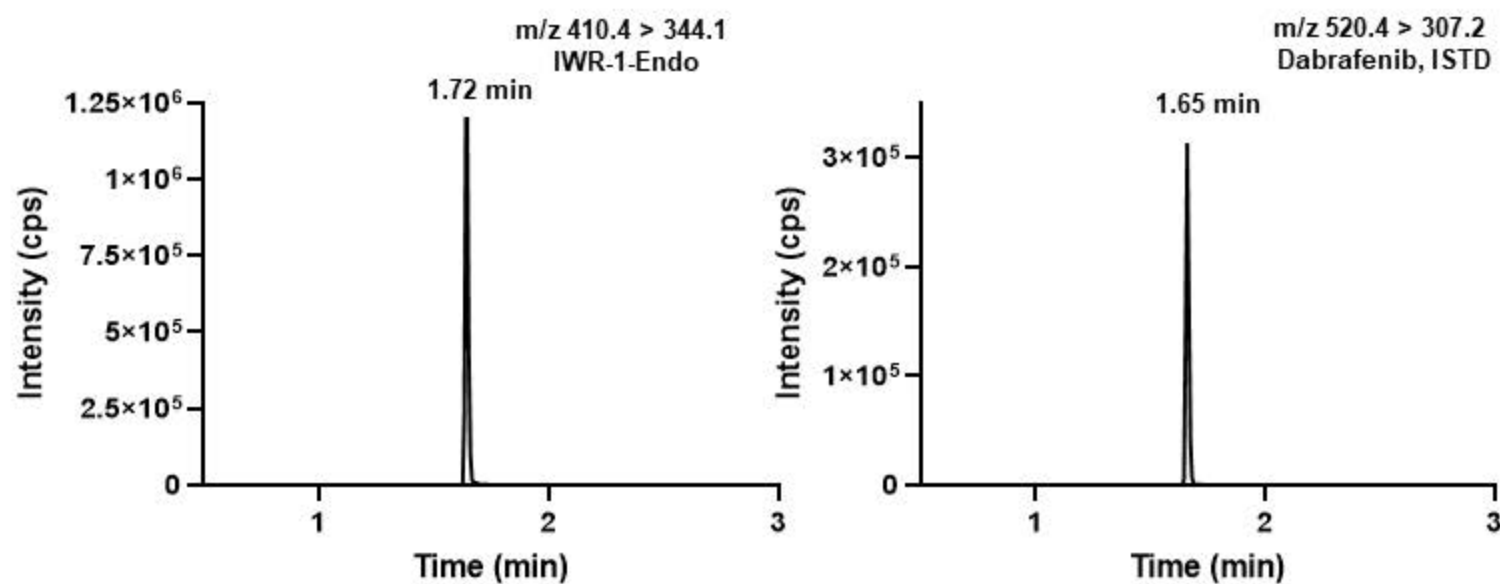

Supplement: Supplementary file 1 [file molecules-27-05448-s001.zip › Supplementary Figure S2.pdf]

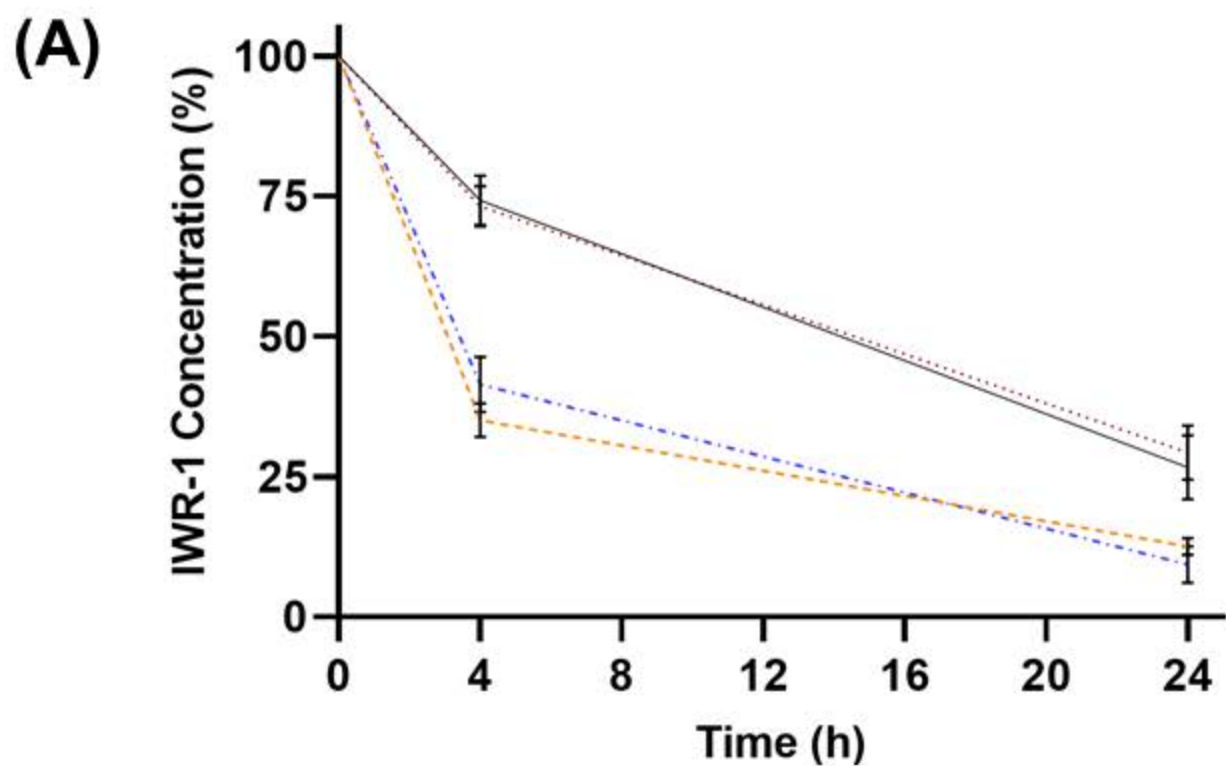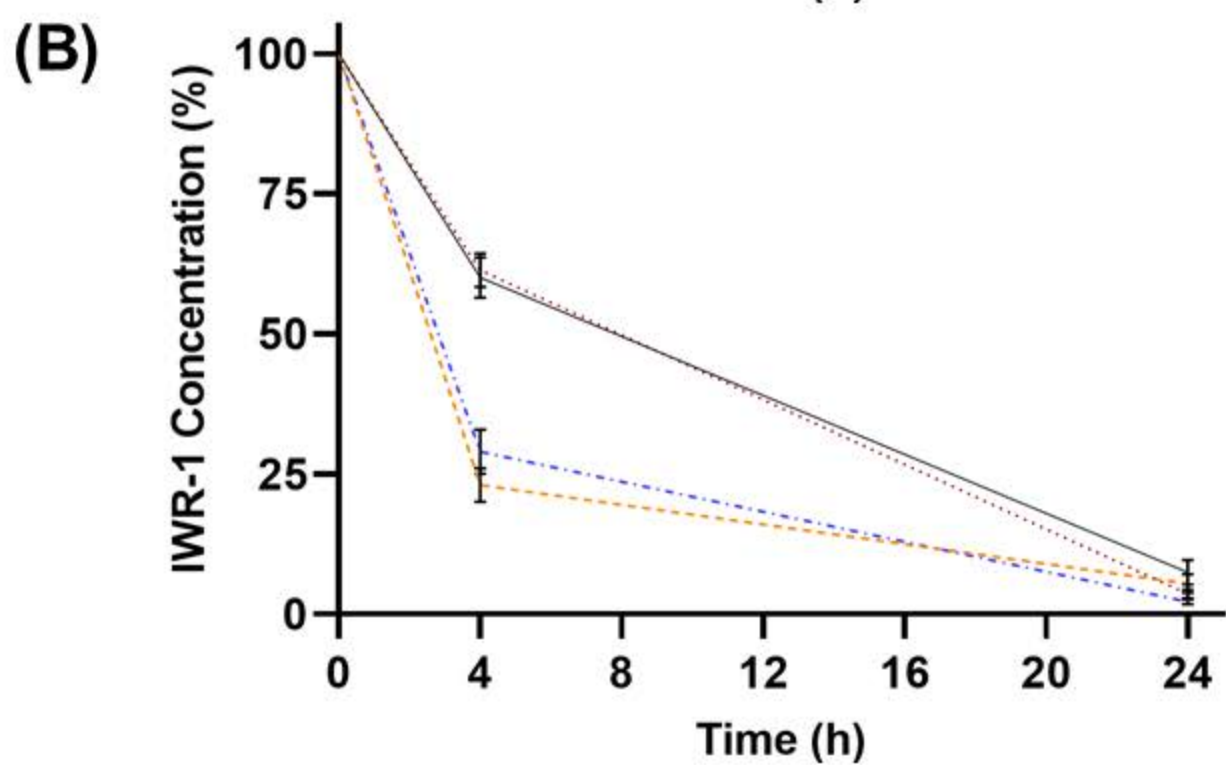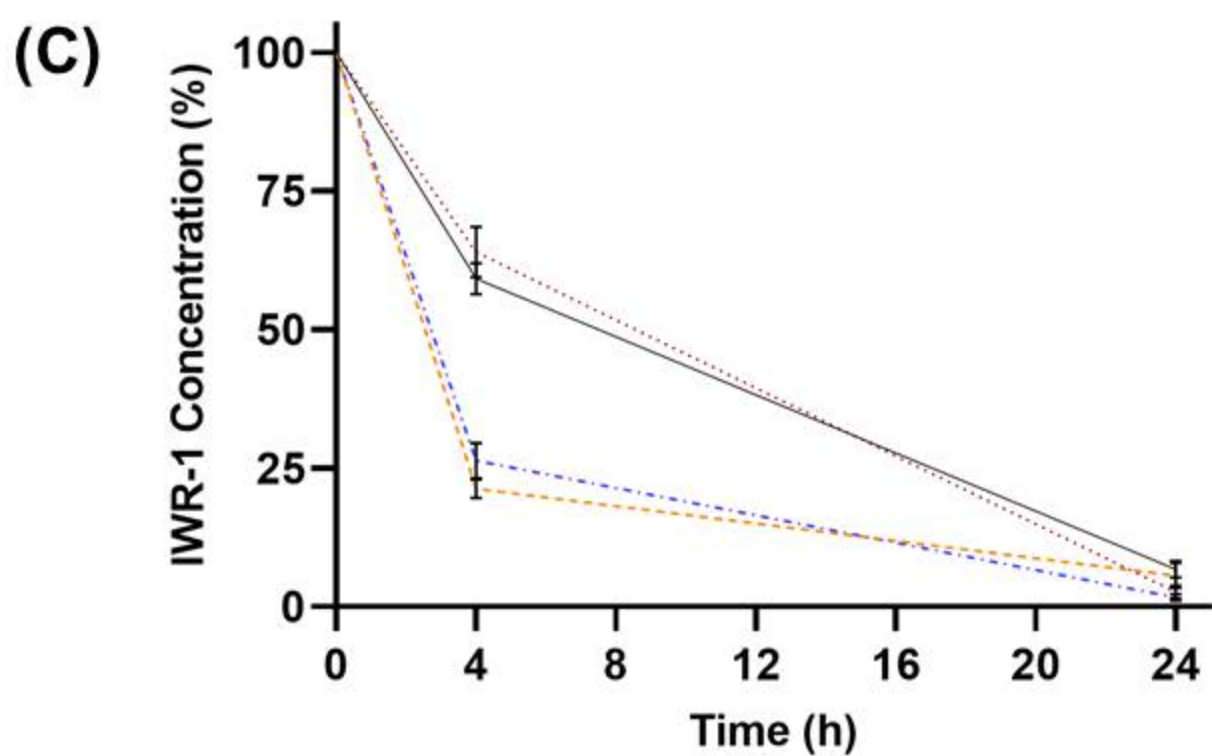

— LQC at 4°C    ..... HQC at 4°C    - - - LQC at RT    - . . HQC at RT

Supplement: Supplementary file 1 [file molecules-27-05448-s001.zip › Supplementary Figure S3.pdf]

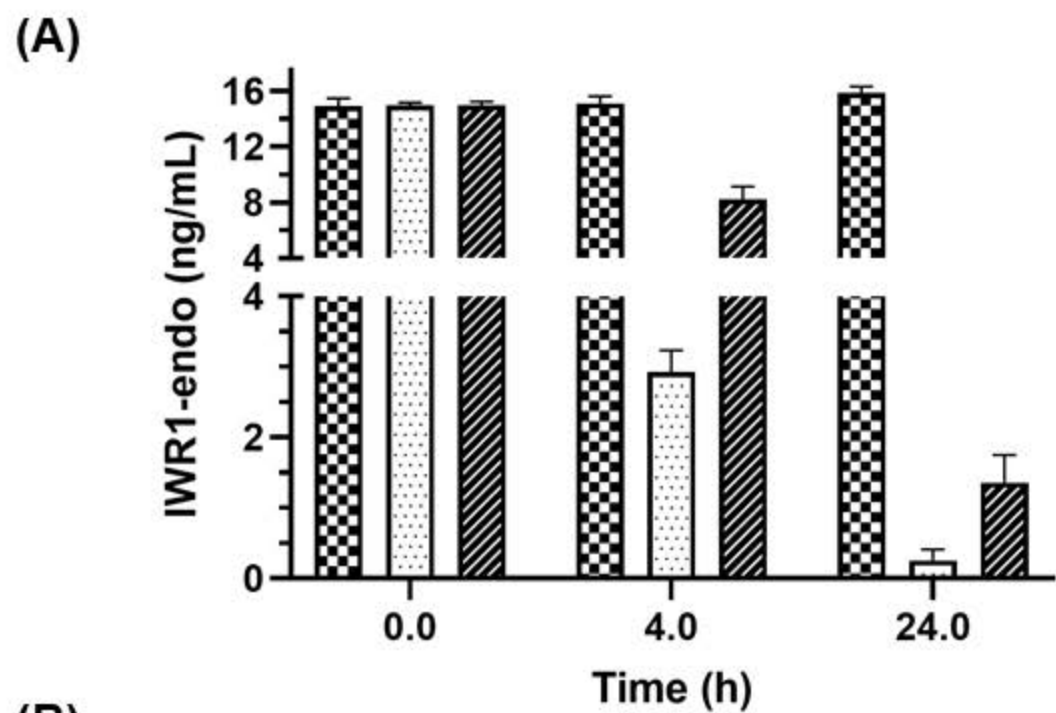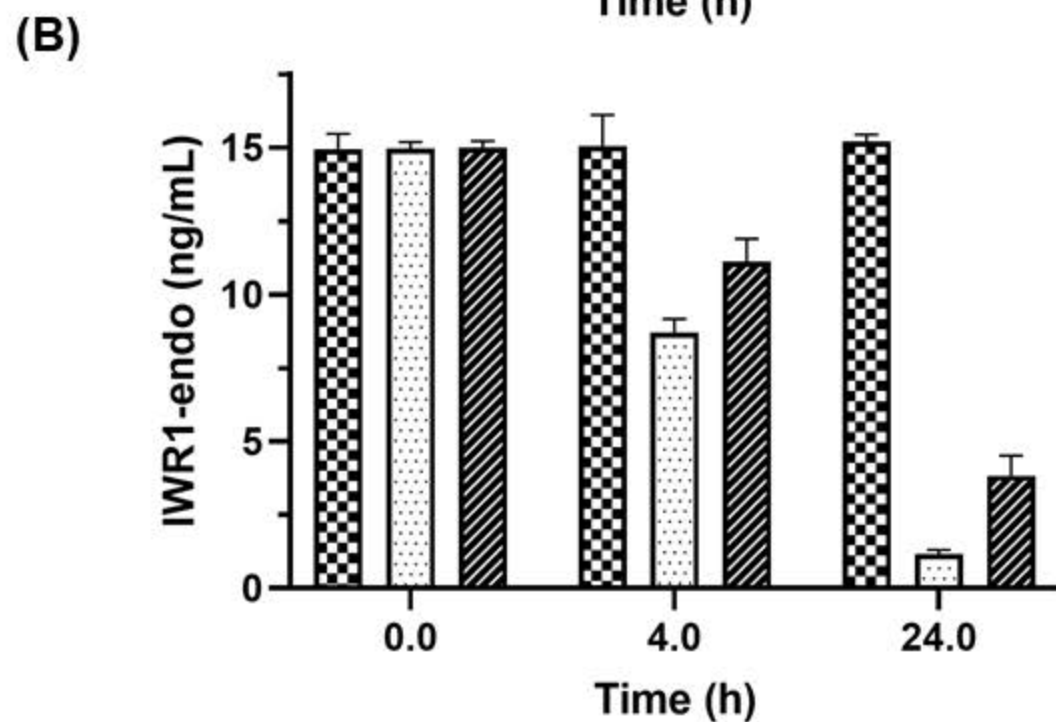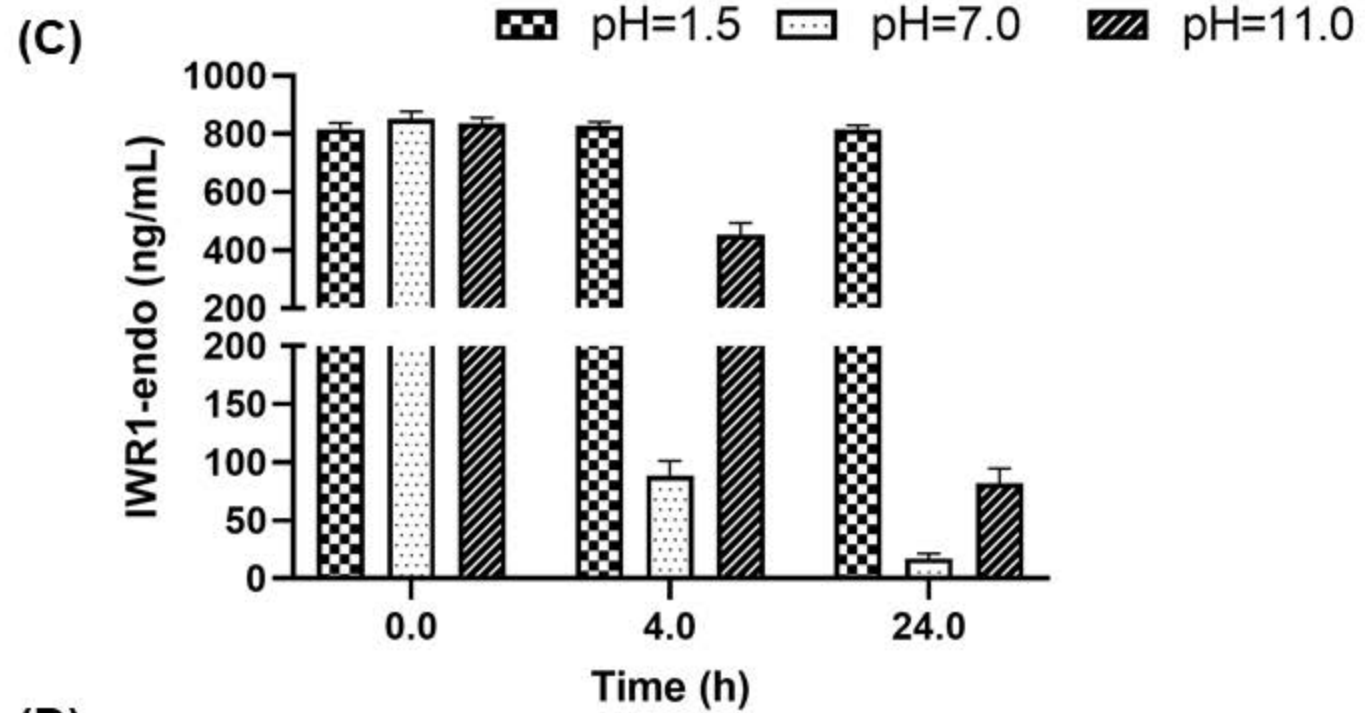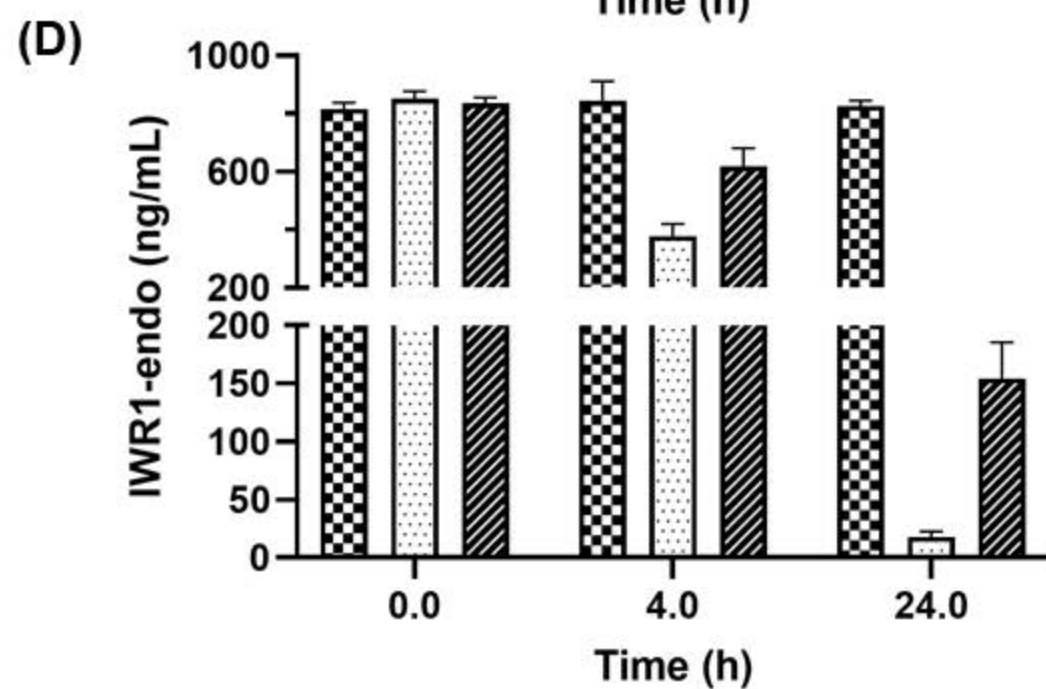

Supplement: Supplementary file 1 [file molecules-27-05448-s001.zip › Supplementary Figure S4.pdf]

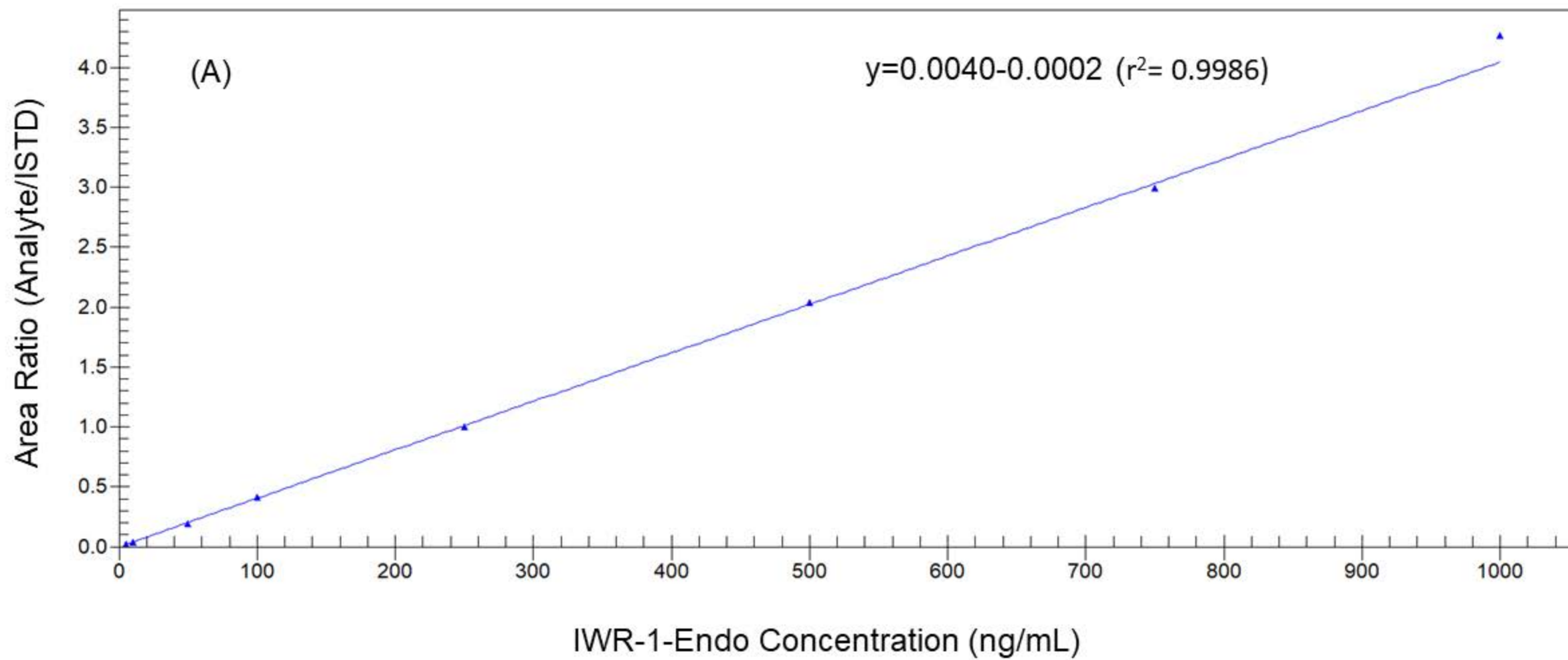

Supplement: Supplementary file 1 [file molecules-27-05448-s001.zip › Supplementary Figure S5A.pdf]

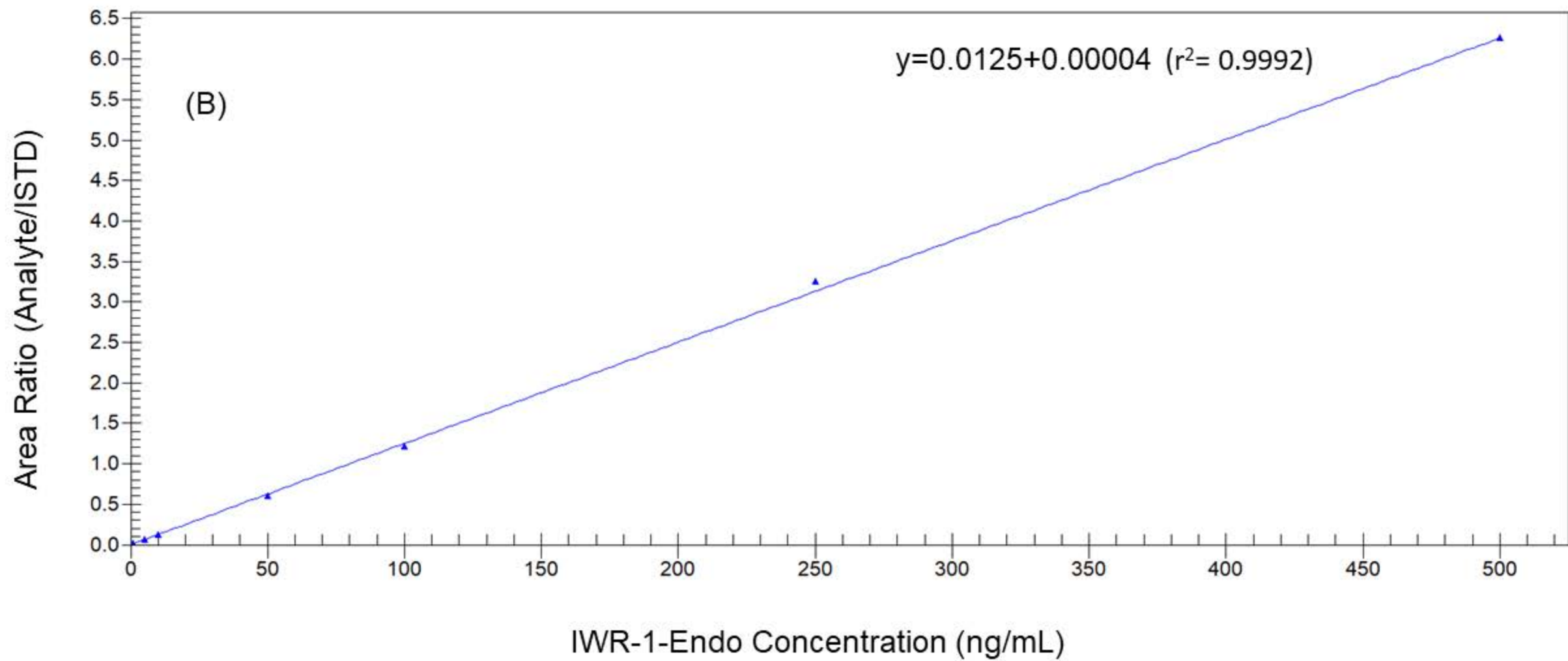

Supplement: Supplementary file 1 [file molecules-27-05448-s001.zip › Supplementary Figure S5B.pdf]
